# An aminostratigraphy for the British Quaternary based on *Bithynia* opercula

**DOI:** 10.1016/j.quascirev.2012.10.046

**Published:** 2013-02-01

**Authors:** Kirsty E.H. Penkman, Richard C. Preece, David R. Bridgland, David H. Keen, Tom Meijer, Simon A. Parfitt, Tom S. White, Matthew J. Collins

**Affiliations:** a BioArCh, Departments of Archaeology & Chemistry, University of York, York YO10 5DD, UK; b Department of Zoology, University of Cambridge, Downing Street, Cambridge CB2 3EJ, UK; c Department of Geography, University of Durham, South Road, Durham DH1 3LE, UK; d Institute of Archaeology and Antiquity, University of Birmingham, Birmingham B15 2TT, UK; e Cainozoic Mollusca, Netherlands Centre for Biodiversity, Naturalis, P.O. Box 9517, 2300 RA Leiden, The Netherlands; f Institute of Archaeology, University College London, 31-34 Gordon Square, London WC1H 0PY, UK; g Department of Palaeontology, The Natural History Museum, Cromwell Road, London SW7 5BD, UK

**Keywords:** Amino acid geochronology, Intra-crystalline protein decomposition, Palaeolithic, Pleistocene, Interglacial

## Abstract

Aminostratigraphies of Quaternary non-marine deposits in Europe have been previously based on the racemization of a single amino acid in aragonitic shells from land and freshwater molluscs. The value of analysing multiple amino acids from the opercula of the freshwater gastropod *Bithynia*, which are composed of calcite, has been demonstrated. The protocol used for the isolation of intra-crystalline proteins from shells has been applied to these calcitic opercula, which have been shown to more closely approximate a closed system for indigenous protein residues. Original amino acids are even preserved in bithyniid opercula from the Eocene, showing persistence of indigenous organics for over 30 million years. Geochronological data from opercula are superior to those from shells in two respects: first, in showing less natural variability, and second, in the far better preservation of the intra-crystalline proteins, possibly resulting from the greater stability of calcite. These features allow greater temporal resolution and an extension of the dating range beyond the early Middle Pleistocene. Here we provide full details of the analyses for 480 samples from 100 horizons (75 sites), ranging from Late Pliocene to modern. These show that the dating technique is applicable to the entire Quaternary. Data are provided from all the stratotypes from British stages to have yielded opercula, which are shown to be clearly separable using this revised method. Further checks on the data are provided by reference to other type-sites for different stages (including some not formally defined). Additional tests are provided by sites with independent geochronology, or which can be associated with a terrace stratigraphy or biostratigraphy. This new aminostratigraphy for the non-marine Quaternary deposits of southern Britain provides a framework for understanding the regional geological and archaeological record. Comparison with reference to sites yielding independent geochronology, in combination with other lines of evidence, allows tentative correlation with the marine oxygen isotope record.

## Highlights

► A stratigraphy using the intra-crystalline protein from the calcitic opercula of *Bithynia* is presented. ► Focused on Britain, but also using samples from a few continental sites, the coverage has been extended back to ∼3 Ma. ► This aminostratigraphy is consistent with independent geochronology, terrace stratigraphy and biostratigraphy. ► The aminostratigraphic framework has been linked to the marine oxygen isotope stratigraphy.

## 1 Introduction

The extent of protein degradation in fossil mollusc shells provides a useful geochronological tool, enabling correlation of fossiliferous deposits and, if calibrated, absolute age estimation (e.g. Mitterer, 1975; Miller and Hare, 1980; Wehmiller, 1982; Murray-Wallace, 1995; Wehmiller et al., 2010). Amino acid geochronology primarily utilizes the slow inter-conversion (racemization) of l-amino acids, the basic building blocks of protein, into an equilibrium mixture of l- and d-amino acids in fossils over time. Previous aminostratigraphic studies in Northern Europe have been based on the epimerization (racemization to a non-mirror-image stereoisomer) of l-isoleucine to d-alloisoleucine (yielding an *A*/*I* value) in the whole shell of molluscs (e.g. Andrews et al., 1979; Miller et al., 1979; Miller and Mangerud, 1985; Bowen et al., 1989; Bates, 1993; Bowen, 1992, 1999, 2000). This pioneering work enabled the development of aminostratigraphies, which could be correlated with the marine oxygen isotope record.

Improvements in sample pre-treatment (Sykes et al., 1995; Penkman et al., 2008a) and new analytical methods (Kaufman and Manley, 1998) have been combined in a series of recent studies. The preparation technique of sample bleaching removes the leachable, open-system matrix of shell protein, leaving a component that exhibits closed-system behaviour (the ‘intra-crystalline’ fraction). The protein degradation in this intra-crystalline fraction is therefore dependent only on time and temperature (Penkman et al., 2008a,b), enabling aminostratigraphic correlation between sites sharing an equivalent integrated temperature history (Wehmiller et al., 2000). Reverse-Phase High Pressure Liquid Chromatography (RP-HPLC) separation with fluorescence detection (Kaufman and Manley, 1998) enables base-line resolution of five amino acid *D*/*L* pairs: aspartic acid, glutamic acid, serine, alanine and valine. The analysis of these amino acids, which racemize at different rates, provides a cross-check on the geochemical integrity of the sample, in addition to yielding isochronic information. As well as racemization, different aspects of diagenesis can be monitored within a closed system, such as the generation of free amino acids and decomposition products. Consequently this approach, which measures the overall extent of intra-crystalline protein decomposition (hereafter IcPD), is significantly different from earlier racemization analyses on British material (e.g. *A*/*I*).

**Fig. 1:**
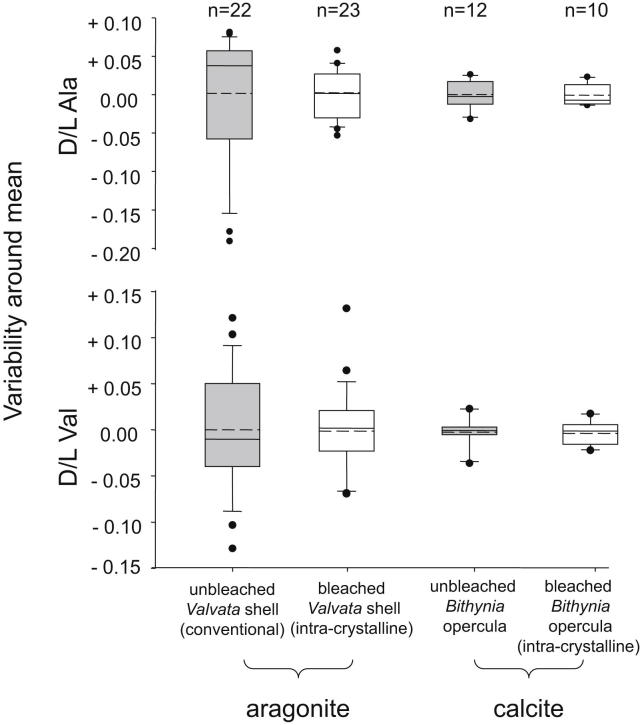
Variability around the mean *D*/*L* of Ala (upper) and Val (lower) for the Total Hydrolysable amino acid (THAA; H*) fraction of unbleached (conventional preparation) and bleached (intra-crystalline) *Valvata piscinalis* shells from Hoxne (data from Penkman et al., 2007) and *Bithynia* opercula. For each site, the base of the box indicates the 25th percentile. Within the box, the solid line plots the median and the dashed line shows the mean. The top of the box indicates the 75th percentile. Where more than 9 data points are available, the 10th and 90th percentiles can be calculated (shown by lines below and above the boxes respectively). The results of each duplicate analysis are included in order to provide a statistically significant sample size. Isolation of the intra-crystalline fraction provides a reduction in the variability observed in *D*/*L* value, and hence a significant improvement in temporal resolution.

Analysis of these five amino acids exclusively from the intra-crystalline fraction of aragonitic gastropod shells has revealed great potential for improved temporal resolution (Penkman et al., 2007). However, the differentiation of sites older than Marine oxygen Isotope Stage (MIS) 9 has been less successful than for younger stages, although the levels of protein decomposition were always shown to be higher using this method. A preliminary set of analyses (Penkman et al., 2007) suggested that calcitic opercula of *Bithynia* had the potential to provide better temporal resolution than had been possible with aragonitic shells (Fig. 6 in Penkman et al., 2007). Since calcite is more stable than aragonite, the possibility that the intra-crystalline proteins are better preserved in this biomineral was explored in relation to the dating of the Hoxnian and Cromerian type-sites. Opercula data from the Hoxnian type-site (MIS 11) showed less natural variability compared to data obtained from *Valvata* shell from the same site (Fig. 1; Penkman et al., 2007; Ashton et al., 2008). Statistical analysis of twelve indicators of protein decomposition at three sites of varying age (including West Runton, the Cromerian type-site) showed that greater temporal resolution was possible using calcitic *Bithynia* opercula than by using aragonitic shells (Penkman et al., 2010).

We built on these preliminary findings by examining a much larger dataset of *Bithynia* opercula and established the consistency of the new IcPD data with existing stratigraphies, particularly those with independent age control (Penkman et al., 2011). In this paper we provide details of the background analyses that underpin the technique. We present results of X-ray diffraction (XRD) analyses of the mineralogy of *Bithynia* shells and opercula, a statistical evaluation of the inter-specific differences between *Bithynia* opercula, and an assessment of the racemization behaviour of different amino acids with time. We present new data from bithyniid opercula from the late Eocene showing persistence of closed system protein for over 30 Ma. The individual analyses for each of the opercula are provided in the (SI) and we review the significance of the results for correlations of Quaternary sequences and Palaeolithic archaeology in an extended discussion.

## 2 Materials and methods

### 2.1 Site selection

**Fig. 2:**
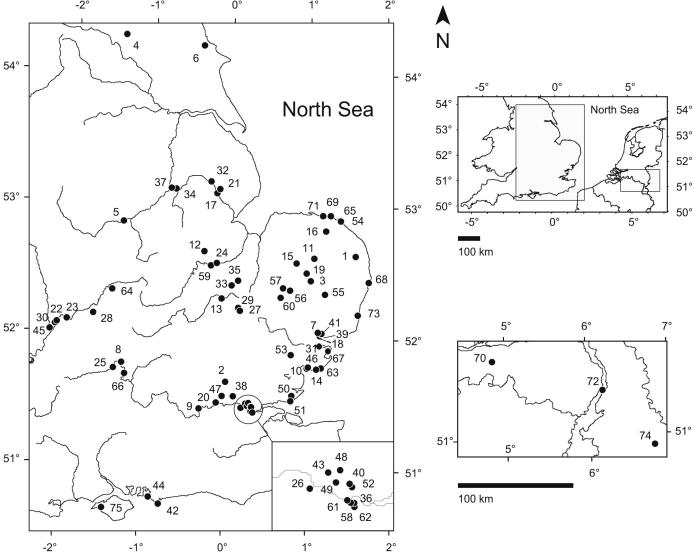
Location map of the sites mentioned in the text from which *Bithynia* opercula have been analysed. The present courses of selected rivers discussed in the text are shown; lowland glaciation, especially in the Middle Pleistocene, will have affected the drainage pattern of these and other rivers.

The sites selected for study (Fig. 2) were chosen because they fulfil one or more of the following criteria:

• They are stratotypes of various interglacial stages.
• They have independent geochronology.
• They can be related to a fluvial terrace sequence.
• They can be related to biostratigraphy.
• They have associated archaeology.

Space precludes detailed information on each site within the main text, but the grid references, bibliographic references and independent evidence of the age of each site is included in the Supplementary online information (SI). The sites fall between 54.12°–50.40° N Latitude and −2.12° W–1.43° E Longitude, within a region with a temperate oceanic climate. Ignoring urban heat effects, almost all sites span a narrow range of mean annual temperature (MAT) of 9–10 °C (Supplementary Fig. 1, adapted from Crown copyright information supplied by the UK Met Office). It is therefore assumed that the samples will have experienced similar integrated thermal histories. In addition to the British sites, we include two stratotypes from the European mainland: the Bavel Interglacial (type Bavelian; Zagwijn and de Jong, 1984) and Tegelen (type Tiglian; Zagwijn, 1960), both in the Netherlands, and Frechen, Germany (van Kolfschoten et al., 1998), which we use as a reference site for the Pliocene.

### 2.2 Materials

An operculum is present in most prosobranch gastropods and is used to close the aperture when the animal withdraws into its shell. Opercula are variable in shape and in chemical composition (Checa and Jiménez-Jiménez, 1998). Some are composed solely of proteins with no mineral support, and consequently rarely fossilize, whereas in others the proteins are mineralized by calcium carbonate (calcite or aragonite), which therefore preserve well in the fossil record.

**Fig. 3:**
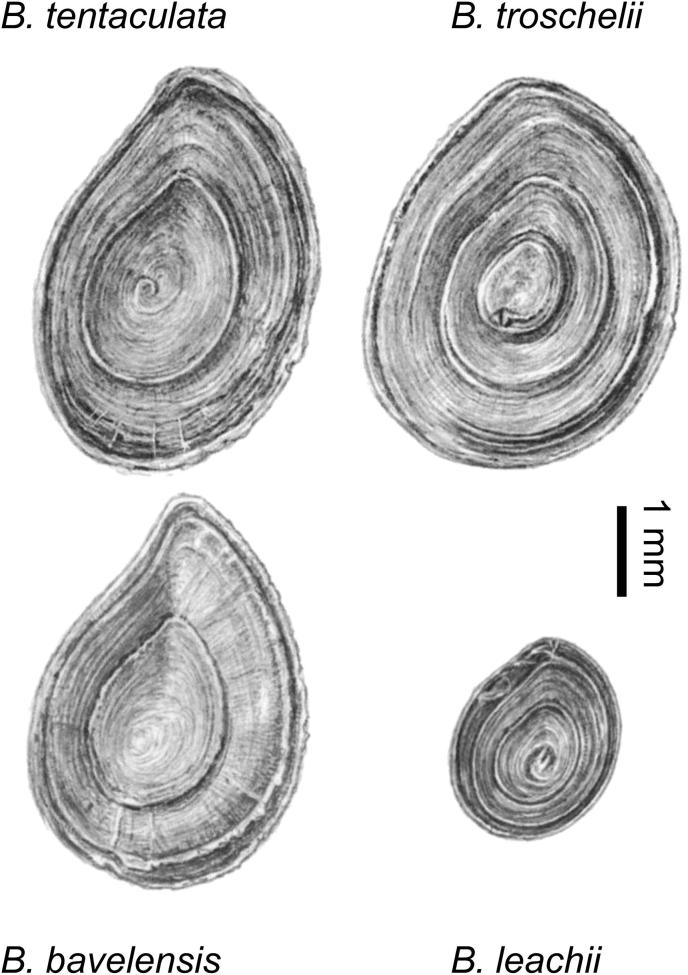
Opercula of *Bithynia tentaculata* (upper left), *B. bavelensis* (lower left), *B. troschelii* (upper right) and *B. leachii* (lower right). Note the more rounded form of *B. troschelii* and *B. leachii*. Images reproduced with permission of Gijs Peeters.

All the opercula analysed here belong to members of the family Bithyniidae, common aquatic gastropods in rivers and lakes, which have a good fossil record in temperate stages of the Quaternary. Species of *Bithynia* are generally absent in sediments deposited during cold stages, although they have been recovered from a few interstadial contexts. Most of our analyses used the opercula of either *Bithynia tentaculata* (Linnaeus, 1758), an abundant species throughout much of Europe, which has an elongate operculum with a pointed apex, or *Bithynia troschelii* (Paasch, 1842), which has a more rounded operculum (Meijer, 1985) (Fig. 3). The name *Bithynia inflata* (Hansén, 1845) has often been used for *B. troschelii*, especially in older English literature. *B. troschelii* is now extinct in Britain but it occurs across central Europe and into eastern Siberia. Opercula from modern specimens of *Bithynia leachii* (Sheppard, 1823), a common European species closely related to *B. troschelii* (both in the subgenus *Codiella*), have therefore been analysed to explore inter-specific differences in protein composition in modern material. *B. bavelensis* Meijer, 1990, an extinct species known only from the late Early Pleistocene (Meijer, 1990; Gittenberger et al., 2004), has an operculum similar in shape to that of *B. tentaculata*, so can only be identified when collected in association with the diagnostic shells.

The only other species used in this study is an Eocene bithyniid, tentatively attributed to ‘*Bithinia conica*’ (Prévost, 1821) by Wood (1877, p. 338), from the Bembridge Limestone, Isle of Wight. This was studied to investigate the survival of amino acids in opercula pre-dating the Quaternary (see Section 3.3).

### 2.3 Methods

We employed the revised technique of amino acid analysis developed for geochronological purposes (Penkman, 2005; Penkman et al., 2008a,b), combining the RP-HPLC method of analysis (Kaufman and Manley, 1998) with the isolation of intra-crystalline amino acids by bleach treatment (Sykes et al., 1995). This combination results in the analysis of *D*/*L* (*dextro*- and *laevo*-rotatory optical isomers) values of multiple amino acids from the chemically protected protein within the biomineral, thereby enabling both decreased sample sizes and increased reliability of the analyses. It should however be noted that there is evidence that the protein within the intra-crystalline fraction of the freshwater bivalve *Margaritifera falcata* can be affected by environmental pH, although this may be due to changes in the aragonitic mineralogy with increased pH or temperature (Orem and Kaufman, 2011).

All samples were prepared using the procedures of Penkman et al. (2008a) to isolate the intra-crystalline protein by bleaching. Two subsamples were then taken from each operculum; one fraction was directly demineralized and the free amino acids analysed (referred to as the ‘Free’ amino acids, FAA, F), and the second was treated to release the peptide-bound amino acids, thus yielding the ‘total’ amino acid concentration (referred to as the ‘Total Hydrolysable amino acid fraction’, THAA). Samples were analysed in duplicate by RP-HPLC. In an attempt to gauge the extent of natural variability in the data, several individual samples were prepared from each horizon. To prevent underestimation of the analytical uncertainty and systematic bias, duplicate samples were routinely analysed on different days (contra the suggestion by Westaway, 2010). The DL ratios of aspartic acid/asparagine (Asx), glutamic acid/glutamine (Glx), serine (Ser), alanine (Ala) and valine (Val) are reported as they are routinely cleanly eluted with baseline resolution.

X-ray diffraction (XRD) analyses (e.g. Cressey and Schofield, 1996) were performed at the Natural History Museum, London, on a selection of bleached powdered opercula and shells from this study, providing information on their crystal structure. Following the methods of Batchelder and Cressey (1998), analyses were undertaken using an Enraf-Nonius PDS 120 X-ray diffractometer, although with tube operating conditions of 35 mA. Silicon powder (NIST SRM 640) and silver behenate were used as calibration standards.

## 3 Results

**Fig. 4:**
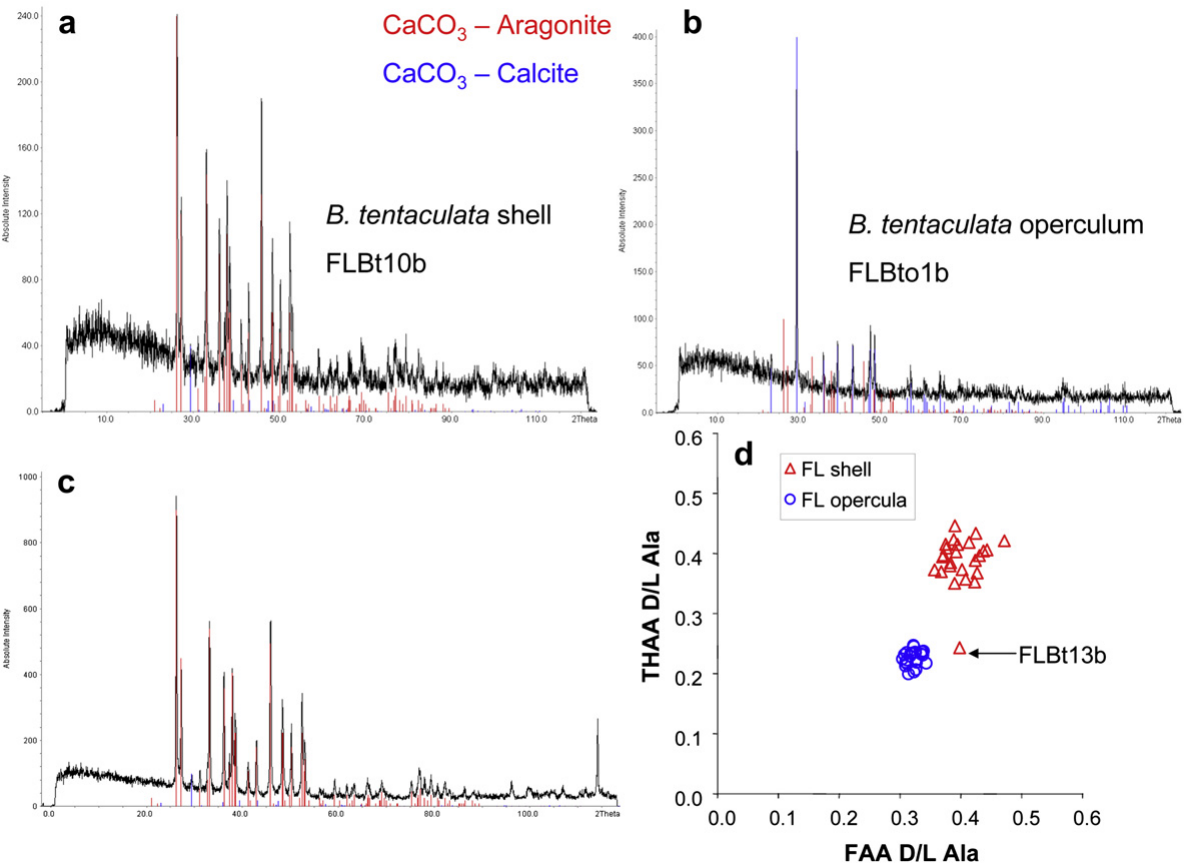
Powder X-ray diffraction analysis of *Bithynia tentaculata* shell (a) and operculum (b) from Funthams Lane East, Peterborough (in black), with reference patterns for aragonite (red) and calcite (blue); the dominant peak of each reference spectrum is scaled to the intensity of the biomineral spectrum. An anomaly in the spectrum for sample FLBt13b (c), a supposedly ‘aragonitic’ *Bithynia* shell, can be observed at 2*θ* = 29.4°, which corresponds to the dominant diffraction peak expected for calcite. This therefore indicates some mineral diagenesis of aragonite to calcite in this shell, which appears to have compromised the closed-system intra-crystalline protein causing it to plot as an outlier (d), modified from Penkman et al. (2007), Fig. 6.

**Fig. 5:**
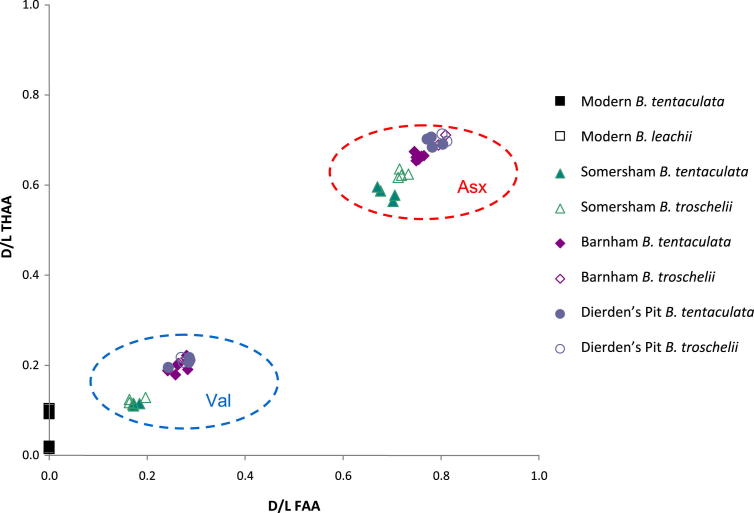
Pairwise comparisons of THAA *D*/*L* vs FAA *D*/*L* for Asx and Val from opercula of *Bithynia tentaculata* and *B. troschelii* of late Middle Pleistocene age (Somersham, MIS 7; Barnham and Dierden's Pit, Swanscombe, both MIS 11) and modern *B. tentaculata* and *B. leachii* from Acle, Norfolk. The data indicate minimal inter-specific variation in *D*/*L* values.

**Fig. 6:**
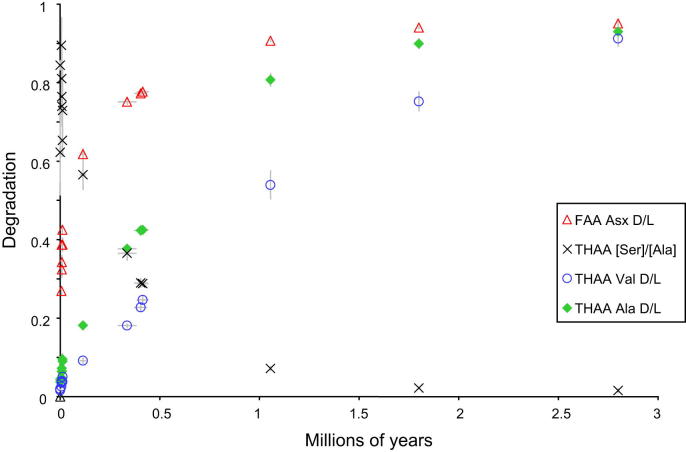
Comparison of racemization in *Bithynia* opercula for FAA aspartic acid (Asx) and THAA valine (Val), alanine (Ala) and [serine]/[alanine] vs time for sites with independent geochronology, showing the benefit of analysing multiple amino acids. Note the rapid racemization in Asx at relatively young sites and the plateauing beyond ∼0.5 Ma; Asx is therefore most valuable for separating sites younger than MIS 7. Val, in contrast, racemizes more slowly and, unlike Asx, is able to differentiate between sites back to the Pliocene, but provides poorer resolution for young sites. The degradation of Ser into Ala also provides a useful chronometer, especially for Middle Pleistocene sites. Utilizing multiple amino acids with different rates of degradation therefore enables greater age resolution. The increase in racemization is not linear with time, but slows during cold stages. *X*-axis error bars are not shown for the Early Pleistocene and Pliocene samples, as these estimates of age are not based on numerical methods.

**Fig. 7:**
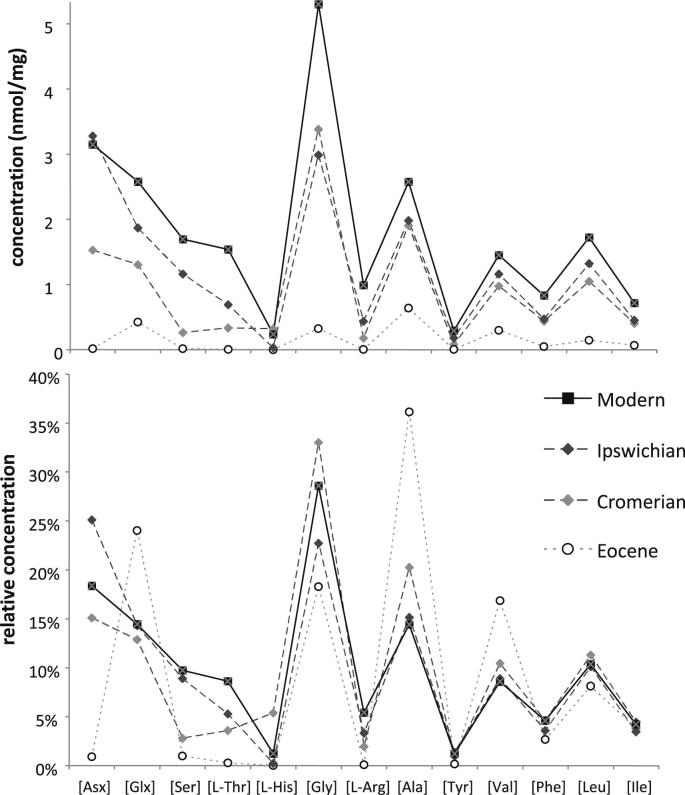
Protein composition of bithyniid opercula from the late Eocene (Bembridge Limestone), type Cromerian (early Middle Pleistocene), the Late Pleistocene (Ipswichian) and modern material, showing degradation of individual amino acids with time. Note the preferential loss of the unstable amino acids (Asx and Ser) and the survival of the most stable amino acids (Glx, Ala and Gly) in the Eocene samples.

**Fig. 8:**
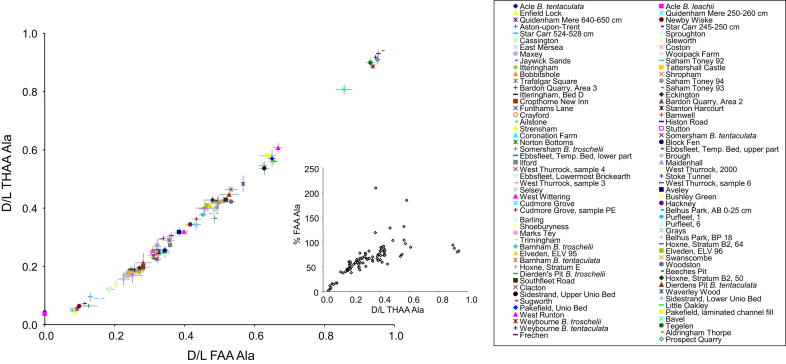
Mean THAA *D*/*L* vs FAA *D*/*L* for alanine in *Bithynia* opercula from sites in Table 1 (*n* = 480). These two measures of breakdown should be highly correlated in a closed-system up to the point where 100% of the amino acids are free; the % FAA Ala vs *D*/*L* Ala is shown in the inset for reference. Error bars represent one standard deviation about the mean for each horizon. Only ∼1% of samples were rejected from calculations of the mean because they do not fall along the expected trajectory. This plot forms an aminostratigraphic framework, where young samples fall towards the bottom left and old samples lie towards the top right of the graph.

**Fig. 9:**
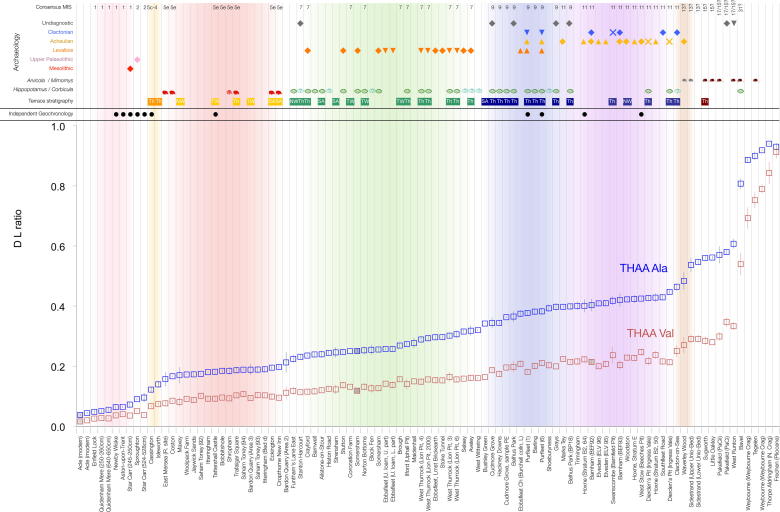
Sites in Table 1, with THAA *D*/*L* Ala and Val plotted against their *approximate* rank order for Ala. Some discontinuities are apparent in the data, which could be attributed to different isotope stages, but in general the data form a continuum, making boundaries between stages difficult to determine. This pattern is expected, since racemization occurs predominantly during temperate stages, with little or no protein breakdown occurring during intervening cold stages (e.g. Miller et al., 1999, Fig. 2). Our data are shown alongside previously published data on terrace stratigraphy (NW: Nene/Welland; SA: Severn/Avon; Th: Thames; TW: Trent/Witham), the occurrence of important biostratigraphic indicator species (indirect association indicated by question mark), associated *in situ* archaeology and existing consensus views on correlation with the MIS record. Age attributions reliant on amino acid dating alone have been excluded. The occurrence of the water vole *Arvicola* is only shown for pre-Anglian sites (i.e. pre-MIS 12). ▲ archaeology found in overlying sediments; ▼ archaeology found in underlying sediments; × archaeology from the same horizon as the opercula analysed; ♦ indirect association (i.e. archaeology recorded from the site but not this profile), or the archaeology occurs both above and below the horizon. *Bithynia tentaculata* (L.) does not occur throughout the British Pleistocene, so where necessary we have used other species of *Bithynia*; at sites where two species are analysed, the shaded symbols indicate the species that is not *B. tentaculata*.

**Fig. 10:**
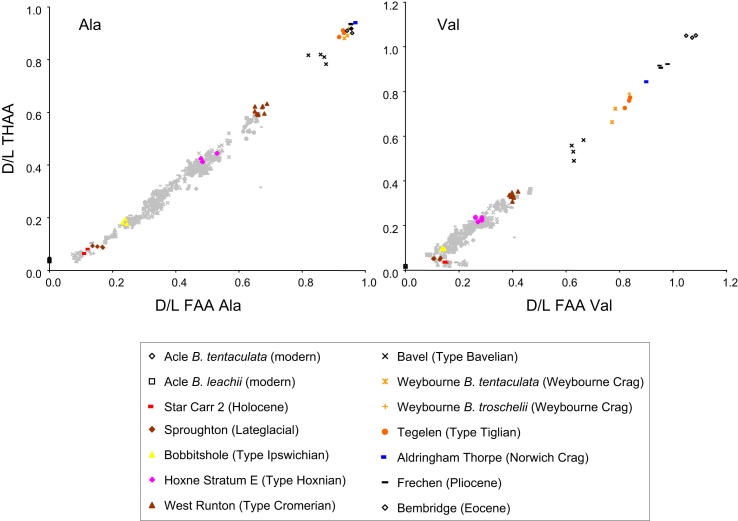
THAA *D*/*L* vs FAA *D*/*L* for alanine (a) and valine (b) in *Bithynia* opercula from sites in Table 1; type-sites and reference sites are highlighted. Star Carr is used as a reference site for the Holocene, Frechen for the Pliocene, and Bembridge for the Eocene. The disposition of data supports the model of more than two interglacial events represented in the British record after the Anglian.

**Fig. 11:**
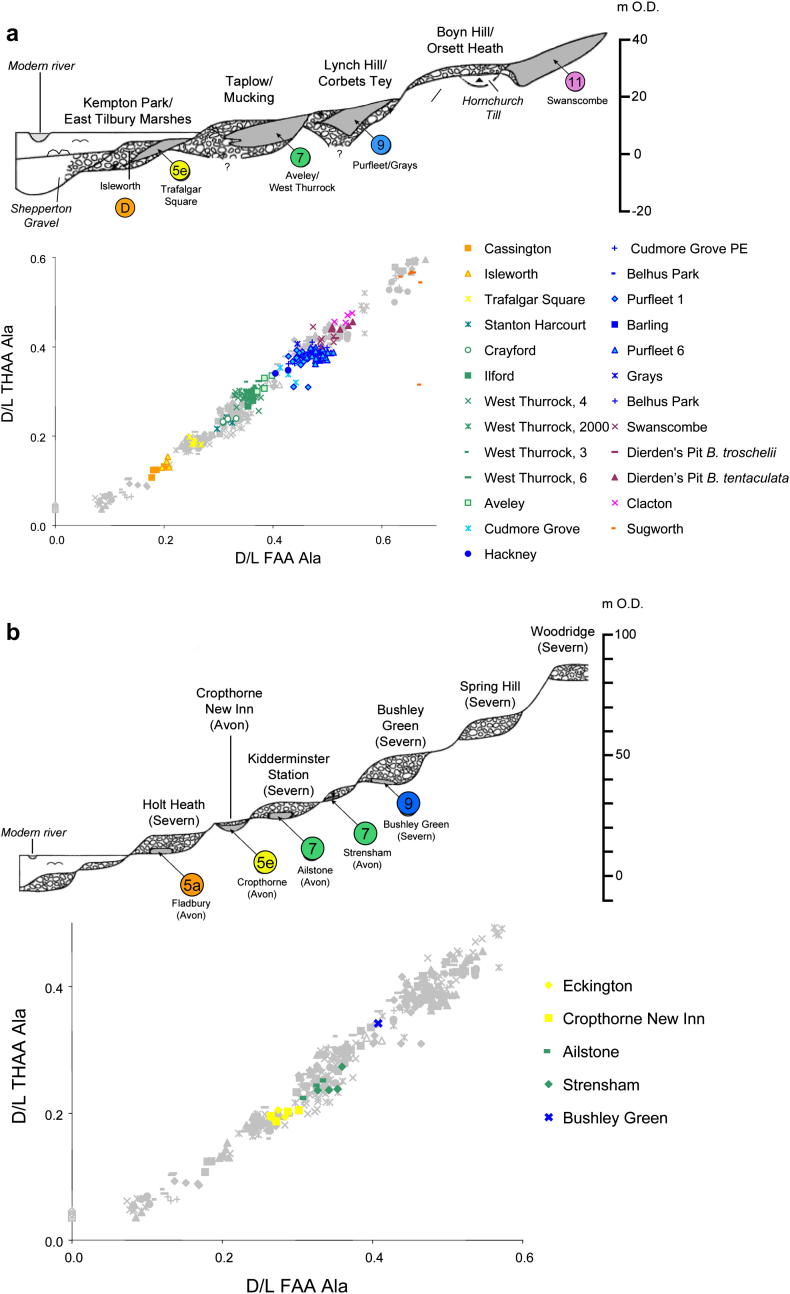

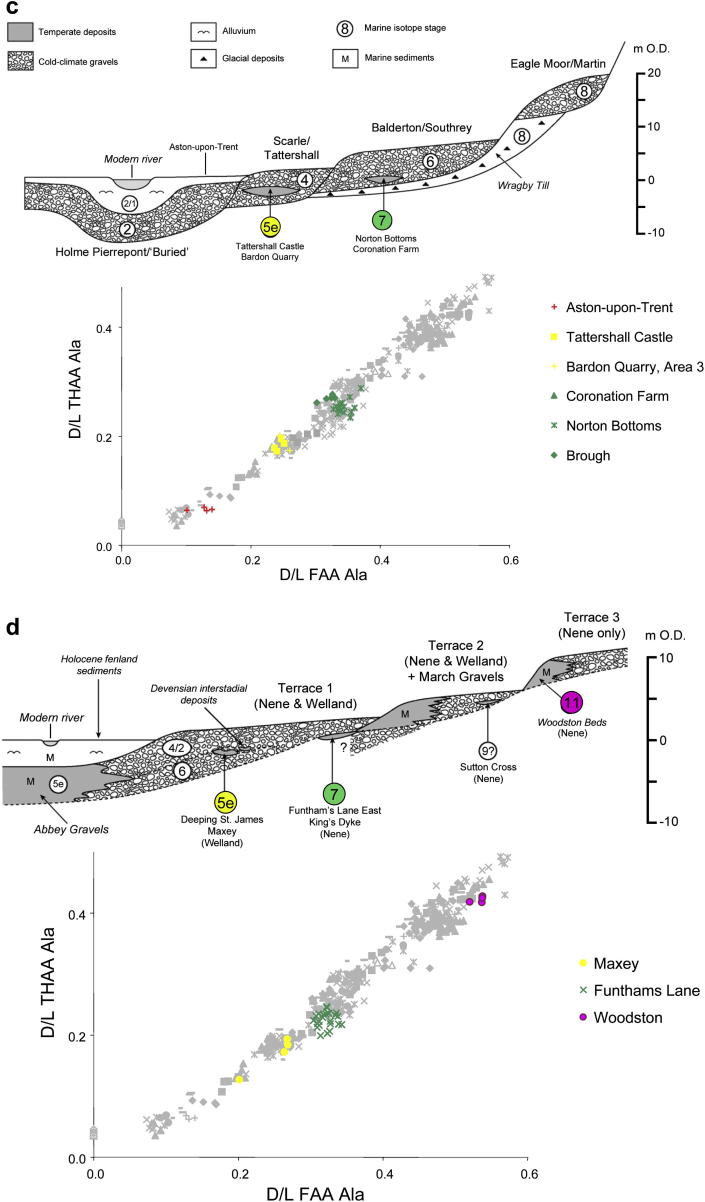
Idealized transverse sections through the terrace sequence and plots of THAA vs FAA *D*/*L* Ala of the (a) Lower Thames (after Bridgland, 2006); (b) Severn/Avon (after Maddy et al., 1991; Bridgland et al., 2004; Bridgland, 2010); (c) Trent/Witham (after White et al., 2007a; Bridgland et al., in press) and (d) Nene/Welland (after Boreham et al., 2010). Note the concordance of relative terrace heights with the extent of protein degradation; higher terraces are older and have more degraded protein within their opercula, although terrace separation in the Nene/Welland (d) is subdued and deposits representing more than one interglacial can be preserved beneath a single terrace surface.

**Fig. 12:**
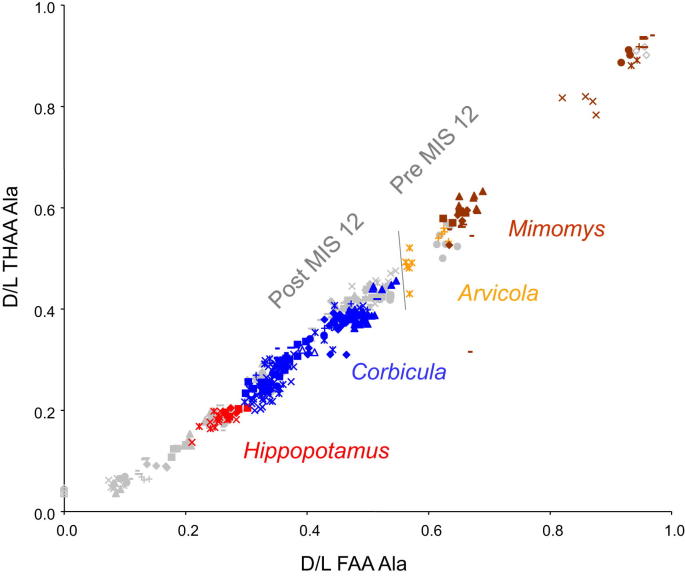
THAA vs FAA *D*/*L* Ala in relation to the occurrence of biostratigraphically important taxa. In deposits after MIS 12, all the sites with *Hippopotamus* fall within a discrete cluster, consistent with the view that they all belong to the Ipswichian (MIS 5e). No sites with *Corbicula* fall within this cluster supporting the view that it is absent from the Last Interglacial but occurs in MIS 7, 9 and 11. In deposits prior to MIS 12, the amino acids are able to discriminate between older sites with the ancestral water vole *Mimomys savini* and younger sites with *Arvicola*, believed to have descended from *Mimomys*. Note that the occurrence of *Arvicola* in many of the post-Anglian sites is not indicated.

**Fig. 13:**
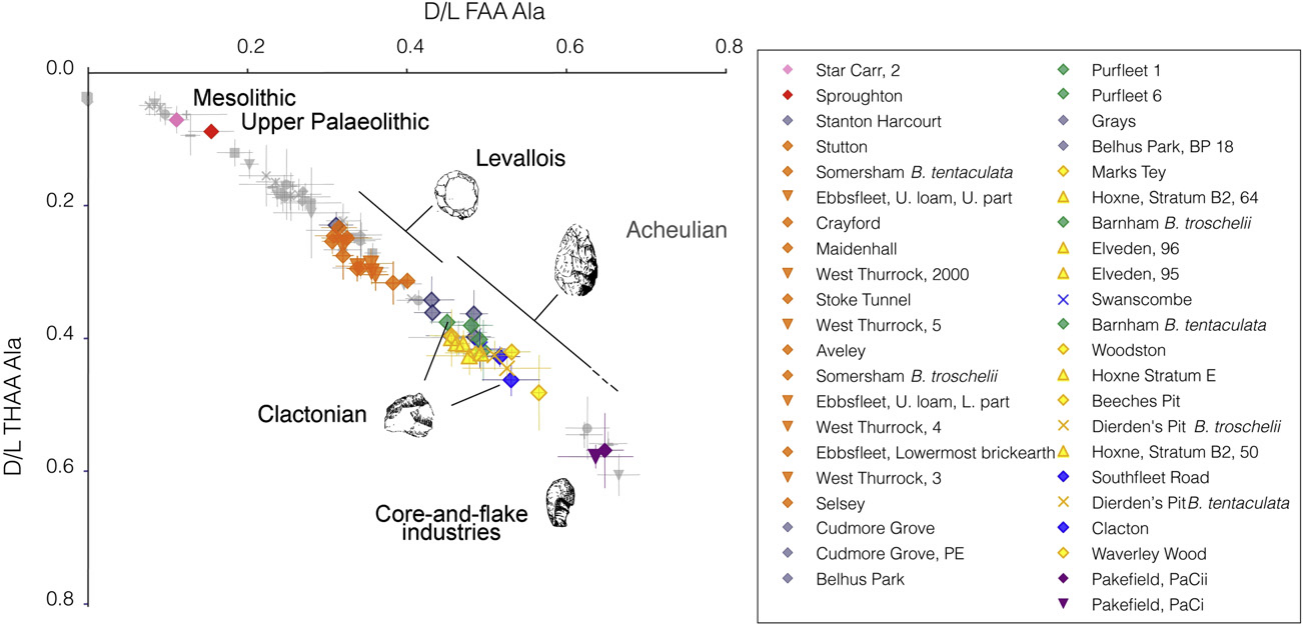
THAA vs FAA *D*/*L* in relation to the occurrence of archaeology (modified from Penkman et al., 2011). Note that the axes are plotted in reverse, so younger samples plot in the upper left hand part of the graph. Amino acid data from pre-Anglian sites yielding Lower Palaeolithic artefacts form two distinct clusters; the oldest (Pakefield) is associated with *Mimomys savini*, the youngest (Waverley Wood) has *Arvicola* (see Fig. 9). Clacton, Southfleet Road and Swanscombe (Lower Loam) are the only localities analysed that have yielded unambiguous Clactonian archaeology (see text); all fall early within MIS 11, although the amino acid data do not enable sufficient resolution within that interglacial to exclude the co-existence of Clactonian and Acheulian industries. However, sites yielding Levallois artefacts form a temporally discrete cluster after MIS 8, supporting the suggestion that the Levallois technique first occurred late during MIS 9. Archaeology has yet to be found at British sites attributed to the Last Interglacial (MIS 5e). *Bithynia* has been analysed from one Upper Palaeolithic (Sproughton) and one Mesolithic site (Star Carr).

**Fig. 14:**
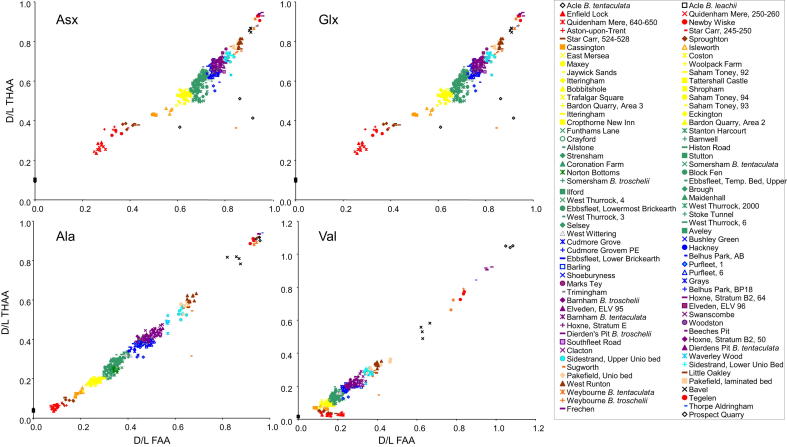
THAA *D*/*L* vs FAA *D*/*L* for the whole dataset of *Bithynia* opercula for aspartic acid (Asx), glutamic acid (Glx), alanine (Ala) and valine (Val). The colouring represents the independent evidence of age for each site (see SI): grey – uncertain, black = modern, red = Holocene, orange = Devensian, yellow = Ipswichian/MIS 5e, green = MIS 7, blue = MIS 9, purple = Hoxnian/MIS 11, light blue & brown = pre-MIS 12 ‘Cromerian complex’.

The results are presented in Table 1, Figs. 4–14 and in the SI, where values for each sample are shown. The data have not been screened and contain a few anomalous outliers (∼1%), which are readily apparent when seen against the whole dataset. Outliers are defined as those values that do not conform to the expected correlation between the THAA and FAA fraction and the relative rates of racemization between the individual amino acids (e.g. Preece and Penkman, 2005). The outliers, which are identified in the SI, have therefore been excluded in the calculation of the means of the data from each site (Table 1, Figs. 8, 9 and 13).

### 3.1 Mineralogy of *Bithynia* shells and opercula

In a study of 59 modern *B. tentaculata* shells, Anadón et al. (2010) found the mineralogy to be purely aragonite and concluded that the presence of a minor calcite component reported in other studies (e.g. Müller, 1968; Leone, 1985) probably resulted from diagenetic and post-depositional changes, or from transformation during sample preparation. We therefore undertook XRD analyses on a small selection of modern and fossil samples (*n* = 21), which have confirmed that the shell of *Bithynia* is made of aragonite, but the operculum is composed of calcite. However, whilst most of the shells analysed were aragonite, outliers from the mean amino acid trends showed some alteration to calcite (e.g. sample FLBt13b in Fig. 4). The peak at 2*θ* = 29.4°, which corresponds to the dominant diffraction peak expected for calcite, should not be present in an aragonitic shell. This therefore indicates some mineral diagenesis of aragonite to calcite in sample FLBt13b (Fig. 4, bottom left). While transformation to calcite during sample preparation cannot be ruled out, post-depositional diagenesis would seem to be a more likely explanation, particularly in view of the anomalous amino acid behaviour (Fig. 4, bottom right). This supports our suspicions that diagenetic alteration within the aragonitic shell mineral affects the intra-crystalline protein fraction.

### 3.2 Extent of inter-specific variation in amino acids from Bithynia opercula

Since no single species of *Bithynia* occurs in all the interglacials of the British Pleistocene, it is important to determine if the opercula of the two common *Bithynia* species used in this study, *B. tentaculata* and *B. troschelii*, have significantly different protein compositions. We therefore analysed the opercula of both species (Fig. 5) from the same horizons from Barnham, Suffolk and Dierden's Pit, Swanscombe, Kent (both MIS 11) and from Somersham, Cambridgeshire (West et al., 1999), a site likely to be MIS 7 in age. *B. troschelii* is extinct in Britain so the closely-related *B. leachii* has been used as a surrogate for the modern sample.

Our analyses show that the opercula of *B. troschelii* yielded slightly different *D*/*L* values to those obtained from *B. tentaculata* from the same horizon, when tested statistically using two-tailed *t*-tests and Mann–Whitney tests (SI). In the samples from Somersham, THAA *D*/*L*s are all slightly higher for *B. troschelii*, although the largest difference is 0.07 (∼9%). In the FAA fraction only the Asx and Glx *D*/*L* are higher but the differences in the means are not large and all are less than 0.04 (<5%). It is in the [Ser]/[Ala] values that the largest difference is observed, where the *B. troschelii* samples have significantly lower ratios. In the Barnham dataset, an increase in *D*/*L* for the *B. troschelii* samples is observed in the Free fractions, where Asx, Glx, Ser and Val *D*/*L* all have higher values, although again the differences are small (not more than 0.05, <13%). In the THAA fraction, only Asx and Val are increased in the *B. troschelii*. The largest differences are observed in the [Ser]/[Ala] values, which are ∼ 0.1 (up to 50%) lower in *B. troschelii*. At Dierden's Pit, *B. troschelii* values are higher only in FAA Asx and Ser and THAA Asx and Val *D*/*L*s. However, in *B. troschelii* the [Ser]/[Ala] values are once again systematically lower. In total around 25% of the statistical tests showed that the proteins in *B. tentaculata* were more degraded than in *B. troschelii* at any one site, while ∼25% showed the reverse and ∼50% showed no statistical difference.

The composition of the protein within the opercula of *B. troschelii* differs slightly from that in *B. tentaculata*, having slightly lower concentrations, most marked in Asx, although the differences are less in the MIS 11 samples. This indicates that the protein composition is different within the opercula of different species from the same genus; consequently the way that the protein breaks down will also be slightly different. However, differences between these two congeneric species are not as marked as those observed in gastropods belonging to different genera (e.g. Penkman et al., 2007). The effect on the overall IcPD is minimal, so the data from these congeneric opercula have been compared directly. The difficulty in identifying *B. bavelensis* and its scarcity in the fossil record precluded us from testing its species effect. However, given these results, we assume in this study that *B. bavelensis* and *B. leachii* will show no major difference in rate of protein breakdown from either *B. tentaculata* or *B. troschelii*. It should be noted, however, that the extent of protein degradation in the opercula of *B. troschelii* tends to be slightly greater than in those from *B. tentaculata*, particularly in younger material.

### 3.3 Nature of protein breakdown with time

If the technique is to be of any use for dating, the first step must be to establish that there is a relationship between the extent of protein decay and age. To do this, we compared our new IcPD data with independent geochronology from the same sites (Penkman et al., 2011). However, few sites in Britain have provided high quality numerical dates, so to assess our amino acid data we have used dates regarded as reasonably reliable by the original authors (SI). Other absolute ages have been obtained from sites in our dataset but have errors that are too large to be useful in this exercise.

The most reliable U-series dates are derived from speleothem material (Smart, 1991), in contexts where *Bithynia* does not occur. Thermoluminescence (TL) has been applied to both sediments and burnt flint (Holyoak and Preece, 1985; Preece et al., 2007). Optically stimulated luminescence (OSL) has become the most widely used method for dating Middle and Late Pleistocene fluvial sediments. However the interpretations of some of these luminescence results have proved problematic because of uncertainties about whether complete zeroing has occurred, difficulties in estimating dose-rates and water content, and from site-specific factors relating to bedrock geology (Briant et al., 2006; Schwenninger et al., 2007). Electron spin resonance (ESR) dating of British Quaternary deposits has proved particularly problematic (Rink et al., 1996) and also suffers from additional difficulties in accurately estimating burial conditions. Radiocarbon dating is the most robust method for dating deposits younger than 40 ka, i.e. the last part of the Last Glacial Stage (MIS 2 and late MIS 3) and the Holocene.

Apart from the Crag material, all of the British Quaternary sites studied here fall within the Brunhes Chron, but palaeomagnetic data are used to constrain the ages of the Reuverian (Late Pliocene) site at Frechen (Gauss) to ∼MIS G17–G19 (∼2.8–3.0 Ma), the type Tiglian at Tegelen (Olduvai) and the Bavel interglacial at Bavel (Jamarillo event). Bavel is thought to correlate with MIS 31, as the magnetostratigraphic boundary within the interglacial is interpreted as the base of the Jaramillo event (Zagwijn and de Jong, 1984). Correlation of the late Tiglian at Tegelen with the marine oxygen isotope record is more problematic. The opercula from the Tiglian stratotype (Pit ‘Egypte’, Tegelen) came from sands that filled a gully incised into clay (Freudenthal et al., 1976), which was of normal polarity according to van Montfrans (1971). The clay has been assigned to pollen stages TC2/TC4b, the gully-fill to TC5, and the overlying deposits to the Eburonian (Zagwijn, 1963). No palaeomagnetic data exist for TC5 at the type locality, but the deposits at the transition from Tiglian to Eburonian are magnetically normal at other Dutch sites (van Montfrans, 1971). According to Lisiecki and Raymo (2005), the first and last interglacials in the Olduvai are MIS 73 and MIS 63. If the palaeomagnetic data have been correctly interpreted, the TC2/TC4b interglacial would have a maximum age of MIS 73, implying that the TC5 interglacial might range in age between ∼MIS 71 and ∼MIS 63. However, mammalian biostratigraphy (Tesakov, 1998; Tesakov, 2004) indicates that the Tiglian type channel-fill contains an arvicolid assemblage considered to belong to zone MNR1, indicating an age earlier in the Matuyama Chron, pre-dating the arrival of the vole *Allophaiomys*, which occurred between 2.1 and 2.0 Ma (Tesakov, 2004; Tesakov et al., 2007).

The *D*/*L* values of aspartic acid/asparagine, glutamic acid/glutamine, serine, alanine and valine (*D*/*L* Asx, Glx, Ser, Ala, Val) provided an overall estimate of protein decomposition (SI). The *D*/*L* of an amino acid will increase over time, but each amino acid racemizes at a different rate, affected by the stability of its peptide bonds and side chain effects.

Asx is one of the fastest racemizing of the amino acids discussed here. It racemizes rapidly as a free amino acid (Liardon and Ledermann, 1986; Smith and Reddy, 1989), but is unusual in that it may also undergo racemization whilst peptide bound (Brennan and Clarke, 1993) via a cyclic succinimide. In contrast, Glx is one of the slower racemizing amino acids. The γ-carboxylate anion should activate racemization, but racemization is slowed by the formation of a lactam (pyroglutamic acid) (see Wilson and Cannan, 1937). This also results in difficulties in measuring Glx in the Free form, as the lactam cannot be derivitized (i.e. labelled) and is therefore undetectable by the machine. Deconvoluting the contribution of asparagine and glutamine to the Asx and Glx *D*/*L* is an added complication (see discussion in Goodfriend and Hare, 1995), but both amino acids have previously been used with success for geochronological studies.

Serine is one of the most geochemically unstable amino acids, producing alanine as one of its decomposition products (Bada et al., 1978). The ratio of the concentration of serine ([Ser]) to the concentration of alanine ([Ala]) therefore gives a useful indication of the extent of protein decomposition within a closed-system, with [Ser]/[Ala] decreasing with time. The *D*/*L* of Ser is less useful as a geochronological tool for samples of Pleistocene age, but is presented here since aberrant values can provide useful indications of contamination.

Alanine is a hydrophobic amino acid and is particularly stable (Conway and Libby, 1958). Its concentration is partly supplemented by the decomposition of other amino acids (most notably serine), which could potentially confound the Ala *D*/*L* signal with time. However, Ala *D*/*L* has been shown to be consistent with relative age in fossil shells (e.g. Wehmiller et al., 2010). Although a slow racemizer, valine is relatively stable, but is not thought to racemize while protein-bound or be a major decomposition product, and is therefore likely to provide a useful marker of age.

The data show an increase in the extent of protein breakdown with time (Fig. 6) in both racemization (*D*/*L*) and degradation of amino acids (e.g. [Ser]/[Ala]). It is important to note that the reaction is temperature dependent and is therefore a non-linear function with time (e.g. Fig. 2 in Miller et al., 1999), with most of the protein breakdown occurring during warm stages. The differing racemization rates are clearly shown in Fig. 6, where Asx is seen to racemize more rapidly than valine (Val). Asx therefore allows better temporal resolution at young sites (less than ∼130 ka), but at older sites, where its values approach equilibrium, the extent of natural variability within the data precludes further age discrimination. Conversely, Val allows only relatively poor age resolution in young contexts but provides much better temporal resolution at older ones, back to the Pliocene. By using a combination of fast- and slow- racemizing amino acids it is therefore possible to discriminate the ages of sites spanning the entire Quaternary. While all the amino acids reported here provide correct relative age information, some are better able to resolve sites of differing age over different stages of the overall breakdown, and therefore it is useful to take into account all of the amino acids when determining age.

To explore the durability of closed-system protein, two opercula of ‘*B. conica’* were analysed from the Bembridge Limestone of the Isle of Wight, dating from the Priabonian Stage of the late Eocene (∼33 Ma; Daley and Edwards, 1990; Hooker et al., 2009). As expected from samples of this age, the amino acids obtained from the intra-crystalline fraction were racemic (*D*/*L* ∼ 1, therefore at equilibrium). The concentration of amino acids was extremely low in these ancient samples, with almost no aspartic acid present (one of the most concentrated amino acids in modern *Bithynia* opercula) and serine and phenylalanine just above that of the background level. As expected, alanine, the most stable amino acids, was the most abundant and was mostly represented in its Free form. The relative abundances of the amino acids in the Eocene samples were of particular interest in demonstrating that the more unstable ones, such as Asx and Ser, were under-represented in comparison with the more stable amino acids, such as Gly and Ala, which dominate the protein content (Fig. 7). Such a signature is predicted from protein decomposition kinetics (e.g. Brooks et al., 1990; Bada et al., 1999) and has also been observed in late Cretaceous mollusc shells (Miller and Hare, 1980). This observation supports the hypothesis that the amino acids isolated in the bleached fraction are original and have not been contaminated by exogenous amino acids. Only 15% of the glutamic acid was in the Free fraction, although as noted above, this is more likely to be a detection issue.

These data show that this indigenous protein exists in samples from as far back as the Eocene. The pattern of amino acid composition opens up the potential for the use of amino acid breakdown as a chronological tool, well beyond the range of the racemization reactions, although the level of temporal resolution at these timescales will be inferior to that obtainable through racemization.

### 3.4 Aminostratigraphic templates for the British Quaternary

Many attempts have been made to refine the temporal resolution and generate numerical dates from kinetic experimental data (see Clarke and Murray-Wallace (2006) for a review), but recent studies have shown that high temperature kinetic experiments may not accurately mimic low temperature protein diagenesis in some biominerals (Demarchi et al., 2012; Tomiak et al., 2012). It has even been proposed that temporal resolution at the level of isotopic substages can be achieved in the Middle Pleistocene using amino acid data (Westaway, 2009, 2010). However, this requires extensive screening of the amino acid data based on individual ratios, in violation of established protocols (cf. McCarroll, 2002), with the potential to lead to under-estimation of the true variability of the data, resulting in a spurious level of resolution and over-interpretation. Such screening procedures are therefore not employed in this study.

Each type of amino acid racemizes at a different rate, but its state (whether it is free or protein-bound) will also determine the extent of racemization. In order to evaluate the overall extent of protein breakdown in a sample, two analyses were undertaken: one of only the free amino acids (FAA), and a second where both the free and peptide-bound amino acids were analysed (the total hydrolysable amino acids, THAA). This provides two measures of breakdown that should be highly correlated in a closed-system, up to the point where 100% of the amino acids are free, when the two fractions are effectively identical. The calculation of concentration (and hence % FAA) is subject to greater error than that of *D*/*L*, as the inherent error in precisely measuring the small volumes and masses involved results in variability in the concentration data, but which cancel out in the calculation of the DL ratios. This, combined with the degradation of free amino acids during the hydrolysis procedure to isolate the THAA, sometimes results in % FAA of greater than 100% (Fig. 8 inset). However, in the majority of samples, % FAA is less than 100%, and therefore departure from the expected FAA *D*/*L* : THAA *D*/*L* relationship indicates a compromised intra-crystalline fraction (e.g. Preece and Penkman, 2005). These two measures, when plotted against each other, provide a relative aminostratigraphic template, where young samples fall towards the bottom left and old samples lie towards the top right of the graph (e.g. alanine in Fig. 8, other amino acid data shown in SI). Rather than defining aminozones, we provided a template showing the behaviour of both modern and fossil opercula as an aminostratigraphic framework for the British Isles (Penkman et al., 2011).

Fig. 9 presents the mean data for THAA Ala and Val *D*/*L* for the sites in Table 1 vs the rank order for Ala *D*/*L* as a proxy for time. These amino acids were chosen as representatives of protein breakdown because Ala is useful for differentiating sites in the Late–Mid Pleistocene, whereas the slow rate of Val racemization informs on the relative age of the Early Pleistocene material. While some inflections in the profile can be related to cold stages (where *Bithynia* is not present), the pattern of data approximates to a continuum. It is therefore difficult to correlate certain sites with specific marine Oxygen Isotope Stages using these data alone, and we have therefore not defined aminozones in this paper. However, in the following section we explore the coherence of the aminostratigraphic framework by reference to other lines of evidence with a bearing on the age of the deposits.

## 4 Discussion: testing the new aminostratigraphy

### 4.1 Comparison with type-sites and/or sites with independent geochronology

In order to interpret the dataset, we initially focused on the type-sites of the temperate stages of the British Quaternary succession (cf. Mitchell et al., 1973; Bowen, 1999) and/or sites with independent geochronology (Fig. 10; see SI for details). In the most recent revised correlation of Quaternary deposits in the British Isles (Bowen, 1999) additional stages were recognized but not defined with formal standard stage names. Instead, the new and existing stages were assigned to a series of ‘aminozones’ linked to a type-site, usually with independent geochronology, or referable to a neighbouring site with such independent age control (Bowen, 1999, Table 2). This approach provided the initial link between the original *A*/*I* aminostratigraphy and the MIS record. Fig. 10 shows the disposition of our new amino acid data for these reference sites against the whole dataset. Sampling is heavily biased towards the Middle and Late Pleistocene, reflecting the focus of our study in these periods compared with the Holocene and the Early Pleistocene. The type-sites at West Runton (Cromerian) and Hoxne (Hoxnian), both in the Middle Pleistocene, and Bobbitshole (Ipswichian) in the Late Pleistocene, are clearly separated, but additional intermediate stages are obviously represented, as suggested by others (e.g. Miller et al., 1979; Bowen et al., 1989; Keen, 1990; Bridgland, 1994; Sutcliffe, 1995).

Early Pleistocene sites in Britain are predominantly marine and occur in the Crag Basin of East Anglia. These ‘crags’ consist of shelly sands and gravels deposited in shallow seas, receiving some input from fluvial sources. These inflowing rivers were the source of occasional non-marine fossils that have been recovered, albeit rarely. *Bithynia* opercula have been analysed from both the Norwich Crag (‘Bramertonian’) at Thorpe Aldringham, Suffolk (West and Norton, 1974; Mayhew and Stuart, 1986), and from the Weybourne Crag at its type locality at Weybourne, Norfolk (Reid, 1882). The Crag material shows the highest levels of protein decomposition of the British Quaternary sites, with Val and Glx supporting the greater antiquity of the Norwich Crag compared to the Weybourne Crag (now included in the Wroxham Crag, see Rose, 2009), consistent with their known stratigraphical relations. When compared to the continental data, the Norwich Crag material shows levels of protein breakdown intermediate between Tegelen (Tiglian type-site) and Frechen (Pliocene), whereas Weybourne Crag material is close to that from Tegelen, again consistent with current thinking (Gibbard et al., 1991).

Geochronological techniques have been applied to some of the British type-sites, not always with satisfactory results. As discussed above, several techniques (e.g. ESR and OSL) can under-estimate true ages, of Pleistocene material, as happened at the Cromerian type-site at West Runton, Norfolk (Rink et al., 1996). The age calculation of ESR dates is heavily dependent on a realistic estimation of the moisture content of the host sediment. Thus ESR dating of tooth enamel from the West Runton mammoth initially produced an age estimate of ∼350 ka (close to the MIS 11/10 boundary), assuming a value of 10% for the moisture content. The age estimate increased to ∼500 ka (MIS 13) using revised moisture content values of 20–30% (Rink et al., 1996), but even these revisions seriously under-estimate what have now been shown to be more reliable ages for the West Runton Freshwater Bed. Biostratigraphical and other lines of evidence now indicate that the type Cromerian does not immediately precede the Anglian (contra West, 1980), assigned to MIS 12, but occurred much earlier within the ‘Cromerian Complex’ (Preece and Parfitt, 2000, 2008, 2012; Preece, 2001), and is probably equivalent to either the early part of MIS 15 or to MIS 17 (Penkman et al., 2010).

The original *A*/*I* dating of West Runton and Waverley Wood, a site at the base of the Baginton Formation in the area of the type Wolstonian succession in Warwickshire (Shotton et al., 1993), also proved problematic. *A*/*I* values on one set of shells from West Runton initially gave anomalously young ages, whereas ratios from Waverley Wood were significantly higher than the ‘accepted’ values from West Runton (Bowen et al., 1989). This led to the suggestion that the Waverley Wood sediments formed during MIS 15, whereas those at West Runton accumulated during MIS 13 (Bowen et al., 1989; Bowen, 1999). Our new amino acid data from *Bithynia* opercula now indicate that West Runton is the older site, a conclusion consistent with the biostratigraphy (Preece and Parfitt, 2000, 2008; Preece, 2001). It is possible that these problems arise from mineral diagenesis of aragonite to calcite, thereby affecting the amino acid geochronology of shell samples over these timescales (Penkman et al., 2010).

Similar problems have been experienced when attempting to date the Hoxnian type-site at Hoxne, Suffolk. Initial U-series/ESR dates on tooth enamel Hoxne yielded a mean age of 319 ± 38 ka, suggesting attribution of the Hoxnian to MIS 9 (Grün et al., 1988). This dating subsequently required significant revision and now indicates that these sediments formed during MIS 11 near the boundary with MIS 10 (Grün and Schwarcz, 2000). This revised date from Hoxne is broadly similar to U-series dates reported from the Hoxnian parastratotype at Marks Tey, Essex (Turner, 1970; Rowe et al., 1999), and from Beeches Pit, West Stow, Suffolk (Preece et al., 2007), which also indicate correlation with MIS 11. A mean TL date of 414 ± 30 ka has also been obtained on burnt flint from the last site, giving even stronger support for an MIS 11 attribution (Preece et al., 2007). Early amino acid data based on *A*/*I* values from shells of *Valvata piscinalis* from the lacustrine sediments (Stratum E) at Hoxne also suggested attribution to MIS 9 (Bowen et al., 1989; Bowen, 1999). However, new amino acid data from *Bithynia* opercula from the lake beds at Hoxne are consistent with an MIS 11 attribution, but the opercula from the upper fluvial sequence (Stratum B2) had lower levels of protein decomposition, making it difficult to determine whether these were of MIS 11 or MIS 9 age (Ashton et al., 2008). A rich vertebrate fauna has also been recovered from Stratum B2 (Stuart et al., 1993), post-dating the lake beds and the ‘Arctic bed’ (Stratum C), which contains several biostratigraphically significant taxa also recovered from Barnfield Pit, Swanscombe. Schreve (2001) assigned this fauna to her ‘Swanscombe mammal assemblage zone’, which she proposed existed during MIS 11. Thus two temperate horizons (Stratum E/D and Stratum B/A2(iii)) occur at Hoxne separated by the ‘Arctic bed’ (Stratum C) interpreted as substages of MIS 11 (Ashton et al., 2008).

Since the Hoxne lake beds (and therefore the Hoxnian) correlate with the early part of MIS 11, no generally agreed British type-site exists for MIS 9. No British site spans the entire period but a number of sites have useful records from this stage. Purfleet is perhaps the most informative site of this age in the Lower Thames valley and has been the site of a number of recent excavations (Schreve et al., 2002; Bridgland et al., 2012). As discussed elsewhere, this site can be tied in to the terrace stratigraphy, and has informative biostratigraphy and archaeology. Original *A*/*I* dating using shells of various species (*Bithynia* and *Corbicula*) from Purfleet proved particularly problematic, suggesting ages that were anomalously old (Miller et al., 1979; Bowen et al., 1989, 1995). These anomalies probably resulted from mineral diagenetic alteration of the mollusc shells, as re-precipitated carbonate occurs throughout the sequence (Schreve et al., 2002; Bridgland et al., 2012). Our new opercula data seem to be immune from such problems and gave values consistent with an MIS 9 age (Fig. 9). Similar amino acid data were obtained from Barling (Bridgland et al., 2001) Cudmore Grove (Roe et al., 2009), Grays and Hackney Downs. Two of these sites have independent geochronology, albeit with relatively large error margins; Purfleet yielded a mean OSL date of 336 ± 47 ka (Bridgland et al., 2012), whereas a mean OSL age of ∼328 ka was obtained from Hackney Downs (Green et al., 2006). Both of these dates are consistent with an attribution to MIS 9.

No formally defined British type-site exists for MIS 7, although the site at Strensham in the Avon valley in Worcestershire (de Rouffignac et al., 1995) has been proposed as the type-site for this particular ‘aminozone’ (Bowen, 1999, Table 2). Our new data (Figs. 8 and 9) show that the extent of protein degradation in the opercula from Strensham is greater than that from the Ipswichian type-site at Bobbitshole near Ipswich, but less than that from the aforementioned MIS 9 sites (Fig. 8). Our amino acid data from Strensham are similar to those from sites such as Aveley, Crayford (Kennard, 1944) West Thurrock (Schreve et al., 2006), Stutton, (Sparks and West, 1964), Uphall Pit at Ilford (Kennard and Woodward, 1900; West et al., 1964) and Stanton Harcourt (Fig. 8). Two of these sites have independent geochronology. Aveley has produced a number of OSL dates consistent with an MIS 7 age (E.J. Rhodes, pers. comm.) and Stanton Harcourt, used as the reference site for MIS 7 by Bowen et al. (1989), has been dated using ESR and U-series, suggesting that the interglacial channel sediments there are at least 147 ka in age (Zhou et al., 1997). These data are therefore broadly consistent with our new amino acid data suggesting correlation with parts of MIS 7 (Fig. 9).

The Ipswichian type-site at Bobbitshole, Suffolk (Sparks, 1957; West, 1957) has no independent geochronology but has yielded fossil assemblages that can be linked to some sites with numerical dates. For example, Tattershall Castle, Lincolnshire, has similar molluscan and plant assemblages and has furnished U-series and TL dates, as well as amino acid data consistent with an MIS 5e attribution (Holyoak and Preece, 1985). Saham Toney, Norfolk, has also yielded preliminary OSL dates broadly consistent with an MIS 5e attribution (R. Briant, pers. comm.). *Hippopotamus*, the classic ‘indicator species’ of the Last Interglacial in Britain (Stuart, 1986; Sutcliffe, 1995), has not been recorded at either Bobbitshole or Tattershall Castle but this almost certainly results from collection failure since extensive collections of vertebrates do not exist from either site. However, *Hippopotamus* is known from the same terrace aggradation as Tattershall Castle at Fulbeck, Lincolnshire (Brandon and Sumbler, 1988), but no molluscs were recovered from these deposits. *Hippopotamus* has also been recovered from several caves, such as Victoria Cave, Settle (Gasgoyne et al., 1981), where flowstones dated by U-series have confirmed an MIS 5e date for the distinctive ‘*Hippopotamus* fauna’.

*Bithynia* is generally extremely rare in cold stage contexts, although it occurred commonly in the interstadial deposit at Isleworth, Middlesex, beneath the Kempton Park terrace of the Thames (Kerney et al., 1982). This deposit yielded a diverse assemblage of thermophilous beetles from a treeless environment, thought to represent the optimum of the Upton Warren interstadial. This was originally thought to fall within the ‘Middle Devensian’ on the basis of an uncalibrated radiocarbon date of 43,140 + 1520/−1280 years BP (Coope and Angus, 1975). The protein from the Isleworth opercula is consistently more degraded than that from Cassington, Oxfordshire, which has been correlated with MIS 5a on the basis of its context, consistent with OSL dates in the range of 80–136 ka (Maddy et al., 1998). The radiocarbon date therefore provides only a minimum age for the Isleworth deposits, the amino acid data indicating an age much earlier in the Devensian, possibly MIS 5c. *B. tentaculata* is known from a number of Devensian Lateglacial contexts, including Star Carr, North Yorkshire (Preece, 1998) and Sproughton, Suffolk (Wymer et al., 1975; Rose et al., 1980). The opercula analysed from Sproughton came from an organic deposit radiocarbon-dated to 12,135 ± 45 years BP. The proteins in these opercula are significantly less degraded than those from Isleworth and Cassington, but exhibit comparable levels of degradation to those from the Lateglacial level at Star Carr.

*Bithynia* is known from many Holocene sites in Britain but we present data from just a few representative localities, which are reasonably well-dated. Perhaps the most famous is the classic Mesolithic site at Star Carr in Yorkshire, which has been the focus of many excavations since its discovery in the early 1950s. A large body of environmental data now exists from this site, which has been subject to a detailed programme of radiocarbon dating (Mellars and Dark, 1998). Our amino acid data indicate that opercula from the Lateglacial horizon (see above) can be distinguished from mid Holocene samples. Analyses from other Holocene sites are all from localities that are well constrained by palynology and/or sites with associated radiocarbon dates. Such localities include Quidenham Mere, Norfolk (Horne, 1999), Enfield Lock, Middlesex (Chambers et al., 1996), Aston-upon-Trent and Newby Wiske (Bridgland et al., 2010). To complete the dataset, we have analysed the opercula from a sympatric population of modern *B. tentaculata* and *B. leachii* from a marsh drain at Acle, Norfolk, from the centre of our study area.

### 4.2 Comparison with fluvial terrace staircases

The formation of Quaternary river terraces in Britain has been shown to be predominantly climate driven, with the phases of aggradation and incision linked to changes in sediment supply and the magnitude of peak discharge resulting from major climate change (e.g. Bridgland and Allen, 1996; Bridgland, 2000, 2010; Maddy et al., 2001; Bridgland and Westaway, 2008). Thus terrace formation by the Thames (Gibbard, 1985, 1994; Bridgland, 1994, 2000), Severn/Avon (Maddy et al., 1991), Trent/Witham (White et al., 2007a) and Nene/Welland (Boreham et al., 2010) is thought to have been driven by glacial–interglacial climatic fluctuation in the Middle and Late Pleistocene, allowing correlations with the MIS record (Bridgland et al., 2004; Rose, 2009). We use these four terrace systems to explore whether our amino acid data are consistent with the terrace stratigraphy (Fig. 11).

The late Middle Pleistocene terrace sequence of the Thames valley is arguably one of the best constrained, in terms of biostratigraphical dating, anywhere in the world, given that it is a valuable repository for mammalian and molluscan faunas, as well as Palaeolithic artefact assemblages (Gibbard, 1985, 1994; Bridgland, 1994, 2000, 2006; Bridgland et al., 2004). Thames terrace deposits extend from the Cotswolds dip-slope to East Anglia, helping tie together the Quaternary sequences in these areas, the stratigraphy underpinned by the effects of the Anglian (MIS 12) glaciation. There has been debate about the attribution of certain sites in the Thames valley to specific marine Oxygen Isotope Stages (Gibbard, 1985, 1994; Bridgland, 1994; Schreve, 2001), but their relative positions within the terrace staircase are not in dispute. The extent of protein decomposition in the opercula from the Thames sites increases with rising terrace elevation (Penkman et al., 2011). Regardless of whether these discrete clusters represent complete isotope stages, substages, or smaller time-spans, they can clearly be discriminated from each other (Fig. 11a).

The oldest fossiliferous deposits in the Lower Thames Valley are those beneath the Boyn Hill/Orsett Heath terrace at Swanscombe, Kent (Bridgland, 1994; Gibbard, 1994). The deposits could be seen in a number of small pits but most have either been worked out (e.g. Rickson's Pit) or are now infilled (e.g. Dierden's Pit). Only Barnfield Pit has been preserved (as a National Nature Reserve) and even here no permanent sections exist. The sequence at Barnfield Pit is complex and consists of a series of fossiliferous horizons (Kerney, 1971; Conway et al., 1996). We present amino acid data from the Lower Loam at Barnfield Pit, which has yielded Clactonian artefacts (Conway et al., 1996; Wymer, 1999), and from the Middle Gravel with Acheulian artefacts from the nearby Dierden's Pit, Ingress Vale (Kerney, 1971). There are large faunal differences between these horizons, especially the appearance of the so-called ‘Rhenish’ molluscs (Kerney, 1971), which suggests that a hiatus exists between the Lower Loam and Middle Gravels. This suggestion gains further support from the occurrence of a palaeosol developed on the surface of the Lower Loam (Kemp, 1985). Notwithstanding these considerations, our amino acid data show similar levels of protein degradation in opercula from both the Lower Loam (Barnfield Pit) and the Middle Gravel (Dierden's Pit), implying that the temporal separation between these deposits cannot have been prolonged; both are likely to have formed during MIS 11.

The sites at Purfleet and Grays in Essex occur within the Lynch Hill/Corbets Tey terrace (Fig. 11). Both sites have yielded rich assemblages of shells and vertebrates, with the addition, at Purfleet, of archaeology. No sections survive at Grays but our sample came from A.S. Kennard's collection in the Natural History Museum, London, and obviously relates to the sections at ‘Grays Thurrock’, which he described at the end of the nineteenth century (Hinton and Kennard, 1900). Purfleet, which like Grays included a number of individual exposures, has been the focus of much recent activity (Schreve et al., 2002; Bridgland et al., 2012). Faunal differences between Purfleet and Swanscombe suggested that these sites belong to different stages (Preece, 1995; Schreve et al., 2002), a conclusion supported by our new amino acid data, which suggest that the sediments at both Grays and Purfleet (and those at Belhus Park, Essex) accumulated during MIS 9 (Fig. 9, Table 1).

Several fossiliferous sites occur in the Taplow/Mucking terrace, including Aveley, West Thurrock and Crayford; all have Levallois archaeology. Our amino acid data suggest that these sites fall within MIS 7. The deposits at Trafalgar Square, which fall within the Kempton Park terrace, are famous for their vertebrate fossils (including *Hippopotamus*) (Stuart, 1986; Sutcliffe, 1995) and molluscs (Preece, 1999) but, in contrast with the earlier Thames sequences, no archaeology has been discovered. These have long been regarded as Last (Ipswichian) Interglacial (MIS 5e) in age, a conclusion supported by our amino acid data. As expected, proteins in the opercula from Isleworth are less degraded that those from Trafalgar Square but show greater deterioration that those from the upstream site at Cassington that has been attributed to MIS 5a (Maddy et al., 1998). As discussed earlier, this suggests that the Isleworth deposits are not ‘Middle Devensian’ as previously thought, but date from a much earlier part of the Devensian.

Our new dataset for *Bithynia* opercula therefore supports the model of four post-Anglian interglacials within the Thames terrace sequence derived initially from terrace stratigraphy (Bridgland, 1994), but supported by amino acid data obtained from both *A*/*I* data in whole shell (Bowen et al., 1989; Bowen, 1999) and from the intra-crystalline protein in shell (Penkman et al., 2007).

The sequence of terraces in the Severn-Avon valley is also well expressed (Maddy et al., 1991) and has yielded aminostratigraphic data consistent with elevation (Fig. 11b). The oldest deposits to have yielded opercula in this valley system are from the Bushley Green Gravel of the Severn (Fig. 11b). The extent of protein degradation is greater in samples from this deposit than in those from either Strensham or Ailstone in the Avon; the Bushley Green opercula are thought to date from MIS 9, an inference that is consistent with the Severn-Avon terrace stratigraphy and with comparison with the more complete record from the Thames (Fig. 11a). Interestingly, there appear to be two terraces separating the Bushley Green Gravel from the New Inn Gravel at Cropthorne, both of which seem to be of MIS 7 age (Fig. 11b), although perhaps relating to different substages. Amino acid data indicate that the deposit at Cropthorne, which has yielded *Hippopotamus* (Maddy et al., 1991) dates from MIS 5e (Fig. 11). One further deposit in this terrace staircase has been dated, namely the Holt Heath Gravel of the Severn, which is thought to have accumulated during MIS 5a (Maddy et al., 1995).

A similar pattern is observable in the terrace sequence of the Trent/Witham system (White et al., 2007a, Fig. 11c). Here the oldest sediments to have yielded opercula are preserved beneath the Balderton-Southrey terrace in Lincolnshire, at Norton Bottoms (White et al., 2007b) and Coronation Farm, Southrey (Penkman, 2007; Fig. 11c). No unheated opercula have been retained from the now inaccessible site at Tattershall Thorpe (Holyoak and Preece, 1985) preserved downstream in equivalent deposits in the right bank tributary Bain system. However, more recent quarrying has exposed sections in both this terrace and a later one at Bardon Quarry, Kirby-on-Bain (White et al., 2007c; see below). The amino acid data from Norton Bottoms and Coronation Farm indicate that these deposits are older than those at Tattershall Castle pit exposed in the next terrace down (Tattershall Castle terrace), suggesting that they formed during MIS 7, whereas those at Tattershall Castle formed during MIS 5e. As discussed earlier, this conclusion is consistent with independent geochronology and other evidence from the site (Holyoak and Preece, 1985).

Terrace separation in the Nene/Welland system is more subdued that those of the other systems discussed, sometimes resulting in the preservation of more than one interglacial beneath the same terrace surface (Fig. 11d). Despite this difficulty, the record appears to be lengthy, extending back to MIS 11, represented by the Woodston beds in the Nene Valley (Horton et al., 1992). No convincing deposits of MIS 9 age are known from either system. Sediments of MIS 7 age are preserved beneath Terrace 1 at Funthams Lane East in the Nene (Langford et al., 2004) and beneath the same terrace in the Welland. MIS 5e deposits occur at Deeping St James (Keen et al., 1999) and Maxey (French, 1982; Davey et al., 1991). One of the opercula from Maxey shows a much younger age; this is one of only two sites where there appears to be a mixed assemblage, the other being Bardon Quarry (Area 2), where one of the opercula analysed indicated an older age than the rest of the samples. The robustness of the opercula makes them more prone to reworking than shells, but only 0.4% of the samples from all of the sites analysed show evidence of this.

### 4.3 Comparison with biostratigraphy

Many of the sites studied have yielded species of biostratigraphical significance. Here we explore the consistency of our new amino acid data with biostratigraphic schemes developed for the following ‘indicator’ species:

#### 4.3.1 Mimomys–*Arvicola* transition

Evolutionary trends in the molar teeth in the water vole lineage are particularly important biostratigraphic markers in the early Middle Pleistocene. Sites that fall early in the ‘Cromerian Complex’ yield the ancestral form *Mimomys savini* with rooted teeth, whereas later sites contain its descendant *Arvicola*, which lack roots (Preece and Parfitt, 2000, 2008, 2012). The *Mimomys*–*Arvicola* transition has been used to rank early Middle Pleistocene sites across Europe in terms of their relative age (von Koenigswald and van Kolfschoten, 1996; Preece and Parfitt, 2000, 2012).

Teeth that grow continuously throughout life without forming roots are an adaptation believed to extend their functional life. Under appropriate environmental conditions, this adaptation is therefore likely to have been under strong selection pressure and will consequently have spread rapidly through the population. Thus it should provide a valuable marker horizon, but the transition is unlikely to have been exactly synchronous throughout the entire range of the voles. New ^40^Ar/^39^Ar dates from Isernia La Pineta in Italy (Coltorti et al., 2005), combined ESR/U-series dates on teeth and infra-red radiofluorescence applied to sand grains from Mauer, Germany (Wagner et al., 2010) suggest that the transition occurred during the early part of MIS 15, a date broadly consistent with its earliest occurrence in NW Europe. Our aminostratigraphic data can now test the prediction of the ‘biostratigraphic age model’ (Preece and Parfitt, 2000, 2008, 2012) that within the early Middle Pleistocene all sites with *Mimomys* are older than those with *Arvicola*. This has important implications for the ‘new glacial stratigraphy’ recently proposed for East Anglia (cf. Preece and Parfitt, 2008, 2012; Preece et al., 2009).

*Bithynia* opercula have been analysed from four pre-Anglian sites (West Runton, Pakefield, Sugworth and Little Oakley) yielding *M. savini* and two sites (Sidestrand and Waverley Wood) yielding *Arvicola* (Fig. 12). Various lines of evidence (Preece and Parfitt, 2000, 2008, 2012; Preece, 2001) suggest that the four *Mimomys* sites are not all contemporary and that the fluvial sediments at Little Oakley, Essex (Bridgland et al., 1990) are slightly younger than the Cromerian type-site at West Runton (West, 1980; Penkman et al., 2010), Pakefield, Suffolk (Parfitt et al., 2005) and Sugworth in the Upper Thames (Briggs et al., 1975). All four sites were believed to be older than the *Arvicola* sites at Sidestrand, Norfolk (Preece et al., 2009) and Waverley Wood, Warwickshire (Shotton et al., 1993). Our new amino acid data (Fig. 12) give independent support for these conclusions, although the small sample size available from Little Oakley does not allow clear differentiation.

#### 4.3.2 Corbicula

In Britain, the occurrence of the bivalve *Corbicula* has long been recognized as indicating warm conditions, although there remains uncertainty as to the precise identity of the species involved (Meijer and Preece, 2000). Before the present stratigraphic schemes had been developed, *Corbicula* was thought to have occurred in both the Hoxnian and Ipswichian interglacials (e.g. Ellis, 1962; Sparks, 1964) but it has become clear that, as well as being absent in the ‘Cromerian Complex’, *Corbicula* was also absent in Britain during the Last (Ipswichian) interglacial (Keen, 1990; Meijer and Preece, 2000). The pattern of occurrence of *Corbicula* in various terrace sequences indicates that it was present during the latter part of MIS 11, in MIS 9 and 7 but not MIS 5e. Thirty-three sites analysed in this study have yielded *Corbicula* (Table 1); one (Dierden's Pit) appears to date from the Hoxnian (MIS 11), ten appear to be of MIS 9 age and eighteen of MIS 7 age. *Corbicula* has not been recovered from any site attributable to MIS 5e, so that our new amino acid data to date support these earlier suggestions (Fig. 12).

#### 4.3.3 Hippopotamus

Unlike *Corbicula*, *Hippopotamus* is known from the early Middle Pleistocene at sites such as Pakefield (Parfitt et al., 2005) and Norton Subcourse (Lewis et al., 2004) in Suffolk, but it does not reappear until the Last Interglacial (MIS 5e), when it became common and widespread, reaching as far north as Durham (Stuart, 1986). *Hippopotamus* is widely regarded as an ‘indicator species’ for the Last Interglacial in Britain (e.g. Stuart, 1986; Sutcliffe, 1995; Schreve, 2001). At Victoria Cave, near Settle in Yorkshire, a flowstone containing the remains of *Hippopotamus* was dated by uranium series to the Last Interglacial (MIS 5e), providing a firm link between the occurrence of *Hippopotamus* and the deep sea record (Gasgoyne et al., 1981). The occurrence of *Corbicula* and *Hippopotamus* in the British Pleistocene is therefore theoretically mutually exclusive and provides a valuable biostratigraphical framework against which to test our aminostratigraphic data. The six post-Anglian sites analysed in this study that have yielded *Hippopotamus* (Table 1) all have levels of protein degradation consistent with an age attribution to MIS 5e, and do not superimpose with any site yielding *Corbicula* (Fig. 12). Thus the amino acid data are fully in support of this biostratigraphic model.

### 4.4 Application: do the amino acid data reveal temporal structure in the British archaeological record?

Having established that the new technique works, we can now ask whether our new amino acid data confirms a temporal structure in the British archaeological record. Until about twenty years ago, there was considerable uncertainty that humans occurred in Britain before the Anglian Stage (MIS 12). The evidence for earliest occupation was based on a few flint artefacts supposedly from beneath till or incorporated within the glacial deposits themselves (see Wymer, 1985 for review). The discovery of Acheulian hand-axes in primary context at Boxgrove, Sussex, dispelled any remaining doubt, although some of the geochronology at the site indicated an MIS 11 age (Bowen and Sykes, 1994), a view at odds with the mammalian biostratigraphy suggesting an MIS 13 age (Roberts, 1994; Roberts and Parfitt, 1999). *Bithynia* does not occur in the archaeological horizons at Boxgrove but our data clearly support the pre-Anglian age for other archaeological sites in southern England, such as Pakefield (Parfitt et al., 2005) and Waverley Wood (Shotton et al., 1993; Lang and Keen, 2005; Keen et al., 2006). Indeed, the data show that human occupation occurred within at least two distinct pre-Anglian stages, the older (Pakefield) associated with *Mimomys* and the younger (Waverley Wood) with *Arvicola*.

An important issue in Palaeolithic archaeology is the contemporaneity or otherwise of various Lower and Middle Palaeolithic industries (cf. Ashton et al., 1994). The Clactonian, a core-and-flake industry initially described from Clacton-on-Sea, Essex (Warren, 1951, 1955; Bridgland et al., 1999), first occurs within Hoxnian sediments representing the earliest post-diversion aggradation of the Thames (Bridgland, 1994). The existence of the Clactonian as a discrete Palaeolithic industry has been much debated (Ashton and McNabb, 1994; McNabb and Ashton, 1995; White, 2000) but a consensus has emerged in support of its distinctness and temporal separation from the Acheulian with its characteristic hand-axes (cf. McNabb, 2007). The only site where unequivocal stratigraphical superimposition of these two industries occurs is Barnfield Pit, Swanscombe, where Clactonian assemblages occur in the Lower Gravel and Lower Loam, and Acheulian assemblages occur from the Middle Gravel upwards (Ovey, 1964; Wymer, 1968, 1985; Conway et al., 1996). At Barnham, Suffolk (Ashton et al., 1998), Clactonian and Acheulian industries have been reported from different parts of the site (Wymer, 1985; Ashton et al., 1994) and the amino acid samples were taken from yet another area of the site (Preece and Penkman, 2005), making it difficult to link these events together. Our amino acid data from Clacton, Swanscombe (Barnfield Pit and Dierden's Pit), Barnham and Southfleet Road (Wenban-Smith et al., 2006) show considerable overlap (Fig. 13) and cannot therefore shed much new light on the debate surrounding the contemporaneity or otherwise of the Clactonian and Acheulian, although further temporal refinement may be possible in the future.

At Purfleet, attributed to MIS 9 (Schreve et al., 2002), the lowermost archaeological assemblage also appears to be Clactonian (Wymer, 1985) but doubt has been cast on this attribution due to the small size of the assemblage (McNabb, 2007). If this material is genuinely Clactonian, it would appear that a similar archaeological succession occurred during MIS 11 and MIS 9, with Clactonian apparently replaced by Acheulian industries (White and Schreve, 2000). Our opercula come from the uppermost of the two horizons containing putative Clactonian artefacts and below those yielding an Acheulian industry (Bridgland et al., 2012); potentially these data thus provide a terminal age for the Clactonian industry in Britain.

The development of Levallois technology, where cores are prepared in a characteristic fashion before the removal of flakes, is not thought to have occurred in Britain before MIS 8 (Bridgland, 1994; White and Ashton, 2003). Knowledge of the archaeology of sites attributed to MIS 9 is not yet sufficient to exclude the possibility of Levallois technology, but tentative evidence from Purfleet suggests its possible deployment during the latter part of this stage (Bridgland et al., 2012). The archaeological record from sites attributed to MIS 7 is far better and shows that Levallois industries replaced the Acheulian during the Middle Palaeolithic (Wymer, 1985, 1999). Our data from sites yielding Levallois artefacts show a tight clustering within MIS 7 supporting this view (Fig. 13).

In recent years it has become clear that humans were not present in Britain during the Last Interglacial (MIS 5e); earlier claims to the contrary have been shown to be based on stratigraphical misinterpretations (Sutcliffe, 1995; Ashton and Lewis, 2002). Archaeological sites such as Aveley, Crayford, Grays, Purfleet, Stutton, Selsey and Stanton Harcourt (Buckingham et al., 1996) that were thought to be of Last Interglacial age have now all been assigned to earlier stages (Figs. 9 and 13). The apparent absence of humans during the Last Interglacial in Britain should be regarded as provisional, since a Levallois assemblage of this age has recently been discovered in primary context at Caours in northern France (Antoine et al., 2006). However, our results confirm that no British site that has yielded archaeology can be attributed to the Last Interglacial (Fig. 13), a conclusion consistent with the notion of a ‘deserted Britain’ during this stage (Ashton and Lewis, 2002; Lewis et al., 2010).

Britain can, however, boast of a rich Upper Palaeolithic record, although this has been largely recovered from caves, in contexts where *Bithynia* does not occur. The Devensian Lateglacial deposit at Sproughton, Suffolk, is the only British site to have yielded *Bithynia* and Upper Palaeolithic artefacts, including a barbed point (Wymer et al., 1975), although the association is not direct. We also provide data from one Holocene archaeological site, Star Carr, the famous Mesolithic site in Yorkshire (Mellars and Dark, 1998).

### 4.5 Complete dataset

In order to interpret the dataset, we colour code other sites where there is independent evidence of age based on biostratigraphy, terrace stratigraphy or other stratigraphical grounds (Fig. 14; see SI for details). The amino acid data provide strong support for the validity of these independent stratigraphical schemes (Fig. 14). It is clear that the different amino acids enable resolution over different time periods. The data show discrete clustering for some marine Oxygen Isotope Stages in some amino acids, but for others there is overlap. Taken together the overall extent of protein degradation therefore provides an integrated signature, but inspection of the behaviour of individual amino acids can lead to enhanced temporal resolution for different parts of the record. For Asx, there is excellent resolution for sites younger than MIS 5, but it is not possible to discriminate between the Early Pleistocene sites using this amino acid. However, both Val and Glx show clear separation between the Early Pleistocene sites, and from the Pliocene material. Ala provides the best discrimination for Middle Pleistocene sites.

For practical use, the extent of protein breakdown in different amino acids from *Bithynia* opercula from UK sites of unknown age can be compared directly with these aminostratigraphic frameworks (Fig. 14). It is recommended that each amino acid and fraction reported here is compared to provide the relative age information, as the individual age correlation from each should support the others; if not, then it is likely that the sample's closed system has been compromised. Calibration is provided by sites yielding independent geochronology, combined with other lines of evidence, which in turn allow tentative correlation with the marine oxygen isotope record.

The bleached intra-crystalline data from the opercula generally support the original non-marine mollusc *A*/*I* framework developed for the UK (Hughes, 1987; Bowen et al., 1989; Bowen, 1999, 2000). For a few sites, earlier *A*/*I* results now appear to need revision. It is noteworthy that even in these cases the problems with AAR dating was either already identified (i.e. Purfleet) or appeared to be the result of diagenetic modification, as observed for the shells from Hoxne. The only instances where there is clear disparity between the results of this and earlier studies relate to ‘Cromerian’ sites, which can only be differentiated using the more diagenetically stable calcitic opercula.

## 5 Conclusions

1. For geochronological purposes, the intra-crystalline protein from the calcitic opercula of *Bithynia* have been shown to provide a consistent dataset, with far less variability than those obtained from analyses of either whole-shell or intra-crystalline protein from aragonitic gastropod shells.
2. Analysis of a range of amino acids that racemize at different rates provides a means of deriving temporally sensitive aminostratigraphic data over longer timescales. For example, aspartic acid racemizes rapidly and is particularly useful for dating relatively young sites (<130 ka), whereas valine racemizes more slowly and is therefore useful for discriminating between older sites (>1 Ma). Alanine is the most useful amino acid for the Middle to Late Pleistocene.
3. Amino acids were also recovered from the opercula of a late Eocene bithyniid (‘*B. conica*’) from the Bembridge Limestone, Isle of Wight. As expected, only the most durable amino acids were present but their survival in such an ancient sample is remarkable, potentially providing a chronological tool well beyond the range of racemization reactions.
4. The protein composition in the opercula of the two most common species of *Bithynia* (*B. tentaculata* and *B. troschelii*) in British Quaternary deposits revealed only minor inter-specific differences. Given that no single species of *Bithynia* occurs throughout the full stratigraphical succession, these results suggest that the aminostratigraphic data obtained for each are comparable.
5. An aminostratigraphic framework is provided for 480 samples from 75 sites (comprising 100 horizons) in southern Britain, ranging from the Early Pleistocene to the present. By using samples from a few continental sites (the type Bavelian, the type Tiglian and the Reuverian (Pliocene) site at Frechen), the aminostratigraphic coverage has been extended back to ∼3 Ma. When FAA and THAA are plotted against each other, the data show a strong correlation.
6. Contaminated or compromised samples are revealed as outliers that plot away from the general trend. XRD analysis of shell samples forming such outliers revealed a composite calcite/aragonite mineralogy, demonstrating that mineral diagenesis is the probable cause of the contamination.
7. The veracity of our aminostratigraphic data has been tested in relation to (a) standard British stratotypes, or (b) other type-sites (not formally defined), and/or (c) those sites with independent geochronology, or (d) sites that can be linked to a terrace stratigraphy, or (e) sites that have associated biostratigraphy.
8. Our new aminostratigraphic data show clear separation of all the standard stratotypes and the informal reference sites designated for other stages.
9. Hoxne and Purfleet, sites that had previously produced problematic *A*/*I* data (Miller et al., 1979; Bowen et al., 1989) and shell IcPD data (Penkman et al., 2007), are now shown to be likely to belong to MIS 11 and MIS 9 respectively.
10. Amino acid data from Isleworth, a site that was previously thought to characterize the optimum of the Upton Warren interstadial, show that it is not Middle Devensian in age but dates from much earlier in the Devensian, possibly from MIS 5c.
11. Fluvial terraces in valleys including the Thames, Severn-Avon, Trent/Witham and Nene/Welland have yielded aminostratigraphic data consistent with terrace elevation.
12. All British sites with the water vole *M. savini* within our aminostratigraphy dataset are shown to be older than pre-Anglian sites with *Arvicola*, giving strong support to the ‘Biostratigraphic age model’ (Preece and Parfitt, 2000, 2008, 2012; Preece et al., 2009) rather than the alternative ‘new glacial stratigraphy’ (Hamblin et al., 2005; Rose, 2009).
13. The aminostratigraphy dataset is consistent with the view that the bivalve *Corbicula* never occurs with *Hippopotamus* in the British Pleistocene (cf. Keen, 1990; Meijer and Preece, 2000), and that within post-Anglian contexts, sediments with *Corbicula* are invariably older than those with *Hippopotamus.* Our aminostratigraphic data from post-Anglian sites yielding either of these species show no superimposition of data points, and strongly support the conclusion that *Hippopotamus* is confined to the Last Interglacial (MIS 5e).
14. Our aminostratigraphic data are consistent with the present understanding of the temporal occurrence of various Palaeolithic industries. The data show that human occupation occurred within at least two distinct pre-Anglian stages, the older (Pakefield) associated with *Mimomys* and the younger (Waverley Wood) with *Arvicola.* Levallois technology is thought to have appeared around the MIS 9–8 transition, although the archaeological record from this interval is poor (Bridgland, 1994; White and Ashton, 2003; cf.; Bridgland et al., 2012). Our results also confirm that no British site that has yielded archaeology can be attributed to the Last Interglacial (Fig. 13), a conclusion consistent with the notion of a ‘deserted Britain’ during this stage (Ashton and Lewis, 2002; Lewis et al., 2010). Amino acid data has been unable to shed much new light on the debate surrounding the contemporaneity or otherwise of the Clactonian and Acheulian, although further temporal refinement may be possible in the future.
15. Using models of terrace formation related to orbital cyclicity (Bridgland, 2000, 2006), combined with biostratigraphy and independent geochronological evidence, our aminostratigraphic scheme can be linked to the marine oxygen isotope stratigraphy for the Late and Middle Pleistocene.
16. Our aminostratigraphic scheme provides a template forming the basis for the interpretation of the dating of other sites from this region. Moreover, the calcitic opercula of bithyniid (or similar) gastropods occur commonly in many Quaternary sequences, offering potential for development and correlation of regional aminostratigraphies around the world.

## Figures and Tables

**Table 1:** Amino acid analyses of the intra-crystalline proteins in the opercula of *Bithynia* from critical sites in southern Britain. Comparative data from three Early Pleistocene/Pliocene sites on the continent are also shown. Sites with independent geochronology are in bold. The sites are listed in **approximate** rank order of age based on the extent of Total Ala *D*/*L*, the most useful single measurement covering the timescales under discussion. Species abbreviations: *b/t*: *B. bavelensis/tentaculata*; *co: B. conica; le*: *B. leachii*; *te*: *B. tentaculata*; *tr*: *B. troschelii*.

Site number (Fig. 2)	*Bithynia* species	Site	No. ind. analysed	Free mean						Free standard deviation						Total mean						Total standard deviation					
				Asx *D*/*L*	Glx *D*/*L*	Ser *D*/*L*	Ala *D*/*L*	Val *D*/*L*	[Ser]/[Ala]	Asx *D*/*L*	Glx *D*/*L*	Ser *D*/*L*	Ala *D*/*L*	Val *D*/*L*	[Ser]/[Ala]	Asx *D*/*L*	Glx *D*/*L*	Ser *D*/*L*	Ala *D*/*L*	Val *D*/*L*	[Ser]/[Ala]	Asx *D*/*L*	Glx *D*/*L*	Ser *D*/*L*	Ala *D*/*L*	Val *D*/*L*	[Ser]/[Ala]
1	*le*	**Acle (modern)**	3	0.00					0.35	0.00					0.19	0.10	0.05	0.08	0.04	0.02	0.62	0.00	0.00	0.01	0.00	0.00	0.11
1	*te*	**Acle (modern)**	2	0.00						0.00						0.10	0.04	0.08	0.04	0.02	0.84	0.01	0.00	0.02	0.00	0.00	0.01
2	*te*	Enfield Lock	4	0.27	0.13	0.42	0.09	0.15	2.28	0.01	0.02	0.01	0.01	0.02	0.33	0.27	0.05	0.24	0.05	0.03	0.90	0.02	0.01	0.01	0.01	0.01	0.07
3	*te*	Quidenham Mere, 250–260 cm	4	0.27	0.23	0.42	0.08	0.19	2.25	0.01	0.10	0.03	0.01	0.03	0.17	0.25	0.06	0.25	0.05	0.03	0.77	0.01	0.00	0.02	0.01	0.00	0.02
3	*te*	Quidenham Mere, 640–650 cm	3	0.28	0.28	0.46	0.09	0.22	2.26	0.02	0.02	0.01	0.01	0.02	0.14	0.26	0.06	0.27	0.05	0.03	0.76	0.01	0.00	0.02	0.01	0.00	0.06
4	*te*	**Newby Wiske**	3	0.34	0.10	0.54	0.10	0.14	1.63	0.02	0.01	0.02	0.01	0.02	0.13	0.34	0.06	0.31	0.06	0.04	0.74	0.01	0.00	0.01	0.01	0.00	0.05
5	*te*	**Aston-upon-Trent**	4	0.32	0.16	0.56	0.12	0.19	1.58	0.02	0.03	0.03	0.03	0.04	0.18	0.36	0.07	0.34	0.06	0.04	0.76	0.01	0.00	0.01	0.00	0.00	0.07
6	*te*	**Star Carr, 245–250 cm**	2	0.39	0.22	0.57	0.11	0.15	1.91	0.00	0.01	0.01	0.01	0.02	0.19	0.36	0.07	0.33	0.07	0.04	0.81	0.01	0.00	0.00	0.01	0.00	0.08
7	*te*	**Sproughton**	4	0.39	0.12	0.64	0.16	0.12	1.44	0.02	0.01	0.02	0.01	0.03	0.07	0.37	0.08	0.36	0.09	0.05	0.73	0.01	0.00	0.05	0.00	0.00	0.11
6	*te*	**Star Carr, 524–528 cm**	3	0.43	0.20	0.63	0.13	0.17	1.38	0.01	0.01	0.03	0.01	0.02	0.07	0.38	0.08	0.37	0.10	0.04	0.65	0.00	0.00	0.01	0.02	0.00	0.04
8	*te*	**Cassington**	4	0.50	0.18	0.82	0.19	0.10	1.09	0.01	0.02	0.01	0.01	0.03	0.09	0.43	0.10	0.51	0.12	0.07	0.67	0.01	0.01	0.02	0.01	0.00	0.07
9	*te*	Isleworth	4	0.57	0.20	0.86	0.20	0.13	1.00	0.01	0.01	0.02	0.01	0.01	0.05	0.45	0.11	0.53	0.14	0.07	0.67	0.01	0.01	0.02	0.01	0.01	0.05
10	*te*	East Mersea, Restaurant	2	0.60	0.21	0.90	0.23	0.12	0.75	0.01	0.02	0.00	0.02	0.02	0.09	0.48	0.12	0.46	0.16	0.08	0.62	0.01	0.01	0.04	0.02	0.01	0.01
11	*te*	Coston	3	0.62	0.20	0.93	0.24	0.14	0.81	0.02	0.01	0.02	0.01	0.02	0.04	0.51	0.13	0.62	0.17	0.08	0.66	0.01	0.00	0.01	0.00	0.00	0.04
12	*te*	Maxey	4	0.60	0.24	0.93	0.25	0.16	0.78	0.05	0.01	0.05	0.03	0.02	0.10	0.49	0.14	0.54	0.17	0.08	0.57	0.04	0.02	0.06	0.03	0.02	0.04
13	*te*	Woolpack Farm	1	0.65	0.17	0.93	0.26	0.17	0.57	0.01	0.02	0.00	0.00	0.00	0.00	0.52	0.13	0.59	0.17	0.09	0.51	0.00	0.00	0.00	0.00	0.00	0.00
14	*te*	Jaywick Sands	4	0.62	0.23	0.92	0.25	0.14	0.71	0.01	0.03	0.01	0.01	0.01	0.07	0.52	0.15	0.58	0.17	0.09	0.59	0.02	0.01	0.05	0.01	0.01	0.04
15	*te*	Saham Toney, 92	4	0.63	0.18	0.92	0.23	0.14	0.67	0.01	0.02	0.01	0.00	0.01	0.04	0.53	0.13	0.61	0.17	0.10	0.57	0.00	0.00	0.02	0.00	0.01	0.03
16	*te*	Itteringham	1	0.65	0.21	0.94	0.27	0.15	0.71	0.01	0.00	0.00	0.00	0.00	0.00	0.50	0.13	0.56	0.18	0.09	0.60	0.01	0.00	0.02	0.00	0.01	0.00
17	*te*	**Tattershall Castle**	4	0.62	0.20	0.94	0.24	0.17	0.73	0.02	0.00	0.01	0.01	0.01	0.05	0.53	0.14	0.61	0.18	0.09	0.57	0.01	0.01	0.02	0.01	0.01	0.04
18	*te*	Bobbitshole	3	0.64	0.20	0.94	0.24	0.14	0.75	0.01	0.01	0.02	0.01	0.01	0.04	0.52	0.13	0.58	0.18	0.10	0.59	0.01	0.00	0.02	0.01	0.00	0.02
19	*te*	Shropham	4	0.64	0.20	0.98	0.27	0.15	0.72	0.01	0.01	0.02	0.01	0.01	0.02	0.53	0.13	0.61	0.18	0.09	0.58	0.01	0.00	0.02	0.01	0.01	0.04
20	*te*	Trafalgar Square	6	0.64	0.19	0.93	0.26	0.14	0.70	0.01	0.02	0.03	0.01	0.03	0.03	0.53	0.13	0.62	0.19	0.10	0.52	0.01	0.01	0.01	0.01	0.01	0.04
15	*te*	Saham Toney, 94	2	0.66	0.16	1.08	0.25	0.16	0.60	0.01	0.01	0.24	0.01	0.02	0.27	0.54	0.12	0.63	0.19	0.11	0.56	0.01	0.00	0.04	0.00	0.01	0.09
21	*te*	Bardon Quarry, Area 3	3	0.63	0.15	0.93	0.26	0.15	0.72	0.01	0.01	0.01	0.01	0.01	0.05	0.52	0.13	0.60	0.19	0.09	0.55	0.01	0.01	0.02	0.02	0.01	0.04
15	*te*	Saham Toney, 93	4	0.65	0.19	0.95	0.27	0.16	0.71	0.02	0.01	0.02	0.02	0.01	0.06	0.54	0.13	0.62	0.19	0.10	0.58	0.01	0.00	0.02	0.01	0.01	0.04
16	*te*	Itteringham, Bed d	4	0.63	0.14	0.94	0.25	0.16	0.68	0.01	0.00	0.01	0.01	0.01	0.05	0.55	0.13	0.64	0.19	0.10	0.53	0.01	0.00	0.01	0.01	0.00	0.03
22	*te*	Eckington	4	0.65	0.20	0.95	0.27	0.16	0.59	0.02	0.01	0.02	0.01	0.01	0.08	0.54	0.15	0.59	0.20	0.10	0.54	0.01	0.00	0.05	0.01	0.01	0.04
23	*te*	Cropthorne New Inn	4	0.65	0.21	0.98	0.28	0.16	0.76	0.01	0.03	0.02	0.02	0.01	0.07	0.54	0.15	0.63	0.20	0.09	0.61	0.01	0.00	0.01	0.01	0.00	0.03
21	*te*	Bardon Quarry, Area 2	4	0.64	0.18	0.94	0.28	0.17	0.64	0.03	0.04	0.02	0.02	0.02	0.09	0.55	0.15	0.65	0.21	0.11	0.55	0.03	0.03	0.05	0.03	0.02	0.06
24	*te*	Funtham's Lane East	25	0.68	0.24	0.97	0.32	0.18	0.55	0.01	0.01	0.02	0.01	0.02	0.04	0.52	0.15	0.62	0.22	0.12	0.51	0.02	0.01	0.06	0.01	0.01	0.04
25	*te*	Stanton Harcourt	4	0.66	0.27	0.97	0.31	0.17	0.68	0.01	0.02	0.01	0.01	0.01	0.03	0.56	0.18	0.64	0.23	0.11	0.56	0.01	0.00	0.02	0.01	0.00	0.03
26	*te*	Crayford	4	0.68	0.28	0.98	0.32	0.17	0.57	0.01	0.01	0.01	0.01	0.01	0.04	0.58	0.18	0.62	0.24	0.12	0.46	0.01	0.01	0.03	0.00	0.01	0.03
27	*te*	Barnwell	2	0.69	0.23	0.99	0.31	0.17	0.54	0.01	0.01	0.01	0.02	0.01	0.01	0.58	0.17	0.65	0.24	0.12	0.47	0.00	0.00	0.02	0.01	0.01	0.01
28	*te*	Ailstone-on-Stour	4	0.67	0.28	0.99	0.32	0.18	0.61	0.00	0.02	0.01	0.01	0.01	0.08	0.57	0.19	0.67	0.24	0.12	0.54	0.02	0.02	0.03	0.01	0.01	0.05
29	*te*	Histon Road	2	0.67	0.23	1.00	0.33	0.18	0.55	0.01	0.01	0.01	0.00	0.00	0.01	0.57	0.17	0.65	0.24	0.12	0.50	0.00	0.00	0.01	0.01	0.01	0.00
30	*te*	Strensham	4	0.68	0.28	1.00	0.34	0.20	0.59	0.01	0.02	0.01	0.01	0.01	0.03	0.54	0.18	0.61	0.25	0.12	0.50	0.01	0.02	0.04	0.02	0.02	0.02
31	*te*	Stutton	4	0.67	0.27	0.98	0.31	0.18	0.49	0.01	0.02	0.02	0.01	0.01	0.03	0.59	0.19	0.66	0.25	0.14	0.44	0.01	0.01	0.01	0.01	0.01	0.03
32	*te*	Coronation Farm	1	0.68	0.32	0.93	0.34	0.19	0.52	0.00	0.02	0.01	0.01	0.01	0.01	0.55	0.19	0.62	0.25	0.13	0.42	0.00	0.00	0.01	0.00	0.00	0.05
33	*te*	Somersham	4	0.69	0.24	0.99	0.33	0.17	0.52	0.02	0.03	0.02	0.02	0.01	0.03	0.59	0.18	0.67	0.25	0.12	0.44	0.01	0.01	0.02	0.01	0.00	0.02
34	*te*	Norton Bottoms	15	0.68	0.26	0.95	0.34	0.19	0.49	0.01	0.03	0.06	0.01	0.01	0.05	0.55	0.19	0.63	0.25	0.13	0.45	0.02	0.01	0.04	0.01	0.01	0.06
35	*te*	Block Fen	4	0.72	0.23	1.00	0.34	0.20	0.49	0.01	0.01	0.02	0.01	0.01	0.02	0.55	0.17	0.63	0.25	0.13	0.45	0.02	0.01	0.03	0.02	0.01	0.02
33	*tr*	Somersham	4	0.72	0.26	0.97	0.31	0.17	0.36	0.01	0.02	0.03	0.01	0.02	0.01	0.63	0.19	0.74	0.26	0.13	0.31	0.01	0.01	0.02	0.01	0.01	0.01
36	*te*	Ebbsfleet, U. Loam, U. part	1	0.68	0.24	0.98	0.32	0.19	0.60	0.00	0.00	0.01	0.00	0.01	0.01	0.59	0.20	0.67	0.26	0.14	0.50	0.00	0.00	0.02	0.01	0.00	0.00
36	*te*	Ebbsfleet, U. loam, L. part	2	0.69	0.27	0.99	0.32	0.19	0.56	0.00	0.02	0.02	0.01	0.01	0.06	0.61	0.20	0.73	0.26	0.14	0.49	0.01	0.01	0.02	0.01	0.01	0.03
37	*te*	Brough	6	0.69	0.24	0.99	0.32	0.20	0.50	0.01	0.01	0.01	0.01	0.01	0.01	0.60	0.21	0.72	0.27	0.16	0.47	0.01	0.00	0.04	0.01	0.01	0.04
38	*te*	Ilford	2	0.70	0.32	0.98	0.36	0.19	0.45	0.01	0.00	0.02	0.01	0.01	0.04	0.60	0.22	0.66	0.27	0.14	0.39	0.03	0.01	0.03	0.01	0.02	0.02
39	*te*	Maidenhall	2	0.71	0.26	1.00	0.32	0.19	0.49	0.00	0.01	0.02	0.01	0.00	0.01	0.60	0.21	0.69	0.28	0.15	0.43	0.00	0.01	0.01	0.02	0.00	0.01
40	*te*	West Thurrock (Lion Pit), sample 4	25	0.71	0.29	0.99	0.36	0.20	0.45	0.02	0.02	0.03	0.01	0.02	0.05	0.62	0.20	0.71	0.29	0.15	0.43	0.02	0.01	0.09	0.01	0.01	0.06
40	*te*	West Thurrock (Lion Pit), 2000	3	0.70	0.28	0.94	0.34	0.18	0.52	0.01	0.01	0.06	0.01	0.01	0.03	0.62	0.20	0.76	0.29	0.16	0.49	0.01	0.01	0.02	0.01	0.00	0.03
36	*te*	Ebbsfleet, Lowermost Brickearth	3	0.71	0.26	0.99	0.34	0.19	0.43	0.01	0.03	0.01	0.02	0.01	0.02	0.63	0.21	0.77	0.30	0.15	0.40	0.02	0.01	0.04	0.01	0.01	0.01
41	*te*	Stoke Tunnel	2	0.71	0.27	0.99	0.34	0.19	0.47	0.01	0.01	0.02	0.01	0.01	0.03	0.61	0.22	0.72	0.30	0.15	0.42	0.00	0.01	0.01	0.01	0.01	0.02
40	*te*	West Thurrock (Lion Pit), sample 3	25	0.72	0.27	0.99	0.36	0.20	0.46	0.01	0.02	0.02	0.01	0.02	0.05	0.63	0.21	0.73	0.30	0.16	0.42	0.02	0.01	0.05	0.01	0.02	0.05
40	*te*	West Thurrock (Lion Pit), sample 6	5	0.71	0.29	0.98	0.36	0.20	0.47	0.01	0.01	0.01	0.01	0.01	0.04	0.62	0.21	0.74	0.31	0.16	0.44	0.01	0.01	0.03	0.01	0.01	0.04
42	*te*	Selsey	3	0.74	0.28	1.01	0.40	0.22	0.39	0.01	0.02	0.01	0.00	0.01	0.04	0.62	0.20	0.66	0.31	0.16	0.40	0.01	0.01	0.02	0.01	0.00	0.01
43	*te*	Aveley	4	0.71	0.29	0.96	0.38	0.19	0.42	0.00	0.02	0.02	0.01	0.01	0.02	0.63	0.22	0.71	0.32	0.16	0.39	0.01	0.01	0.02	0.02	0.01	0.02
44	*te*	West Wittering	3	0.74	0.30	1.03	0.40	0.21	0.38	0.01	0.01	0.03	0.01	0.01	0.05	0.62	0.22	0.74	0.32	0.16	0.37	0.01	0.01	0.05	0.00	0.00	0.07
45	*te*	Bushley Green	1	0.72	0.40	1.01	0.41	0.24	0.40	0.00	0.00	0.01	0.00	0.01	0.01	0.64	0.28	0.68	0.34	0.16	0.37	0.00	0.00	0.00	0.00	0.00	0.01
46	*te*	Cudmore Grove	4	0.75	0.25	1.01	0.43	0.23	0.41	0.00	0.01	0.01	0.01	0.01	0.01	0.64	0.22	0.72	0.34	0.19	0.40	0.02	0.01	0.02	0.02	0.01	0.01
47	*te*	Hackney Downs	2	0.74	0.27	1.01	0.42	0.24	0.42	0.01	0.00	0.00	0.01	0.01	0.02	0.62	0.21	0.72	0.34	0.17	0.40	0.01	0.00	0.01	0.01	0.01	0.00
46	*te*	Cudmore Grove, sample PE	2	0.75	0.29	1.01	0.43	0.26	0.37	0.01	0.02	0.06	0.01	0.01	0.07	0.64	0.26	0.68	0.36	0.20	0.41	0.00	0.01	0.01	0.00	0.00	0.03
48	*te*	Belhus Park	1	0.77	0.44	0.90	0.49	0.30	0.25	0.00	0.01	0.04	0.01	0.01	0.05	0.59	0.28	0.55	0.36	0.20	0.23	0.00	0.01	0.13	0.02	0.02	0.2
36	*te*	Ebbsfleet, Lower Brickearth	3	0.75	0.33	0.99	0.42	0.24	0.34	0.01	0.03	0.04	0.02	0.01	0.06	0.69	0.29	0.81	0.37	0.21	0.34	0.02	0.01	0.01	0.01	0.01	0.04
49	*te*	**Purfleet, 1**	23	0.75	0.28	1.02	0.45	0.24	0.36	0.01	0.03	0.01	0.01	0.01	0.03	0.66	0.25	0.80	0.38	0.18	0.37	0.01	0.01	0.03	0.01	0.01	0.02
50	*te*	Barling	4	0.77	0.37	1.00	0.48	0.30	0.38	0.01	0.01	0.08	0.01	0.02	0.02	0.66	0.27	0.75	0.38	0.20	0.35	0.01	0.01	0.02	0.01	0.01	0.04
49	*te*	Purfleet, 6	24	0.76	0.33	0.99	0.48	0.26	0.37	0.01	0.02	0.03	0.01	0.01	0.03	0.64	0.25	0.74	0.38	0.21	0.37	0.01	0.01	0.03	0.01	0.01	0.03
51	*te*	Shoeburyness	5	0.77	0.33	0.92	0.49	0.28	0.34	0.01	0.05	0.09	0.01	0.01	0.02	0.64	0.26	0.67	0.39	0.20	0.34	0.01	0.01	0.05	0.01	0.01	0.02
51	*te*	Shoeburyness	1	0.77	0.24	1.01	0.48	0.29	0.34	0.00	0.00	0.01	0.00	0.00	0.00	0.64	0.24	0.63	0.37	0.20	0.33	0.00	0.00	0.02	0.00	0.01	0.01
52	*te*	Grays	2	0.75	0.29	1.03	0.46	0.24	0.36	0.01	0.04	0.02	0.02	0.04	0.05	0.69	0.25	0.74	0.40	0.20	0.33	0.02	0.01	0.05	0.01	0.00	0.07
53	*te*	Marks Tey	2	0.80	0.28	1.03	0.46	0.27	0.26	0.01	0.02	0.03	0.01	0.00	0.01	0.74	0.26	0.84	0.40	0.22	0.24	0.03	0.03	0.09	0.01	0.01	0.01
48	*te*	Belhus Park, BP18	4	0.77	0.36	1.04	0.49	0.28	0.31	0.01	0.01	0.02	0.01	0.01	0.01	0.65	0.26	0.72	0.40	0.21	0.30	0.02	0.01	0.03	0.01	0.01	0.02
54	*te*	Trimingham	13	0.76	0.28	0.98	0.47	0.29	0.30	0.02	0.03	0.05	0.02	0.02	0.03	0.67	0.25	0.73	0.40	0.22	0.29	0.02	0.01	0.07	0.01	0.01	0.04
55	*te*	**Hoxne (2001 (64); Stratum B2)**	4	0.76	0.33	1.03	0.46	0.27	0.30	0.01	0.02	0.01	0.03	0.01	0.01	0.69	0.30	0.79	0.40	0.22	0.30	0.01	0.01	0.02	0.02	0.01	0.01
56	*tr*	Barnham, BEF92	3	0.80	0.40	1.04	0.49	0.28	0.22	0.01	0.04	0.03	0.00	0.01	0.01	0.71	0.28	0.71	0.40	0.21	0.23	0.01	0.02	0.03	0.02	0.01	0.01
57	*te*	Elveden, ELV 96	2	0.77	0.40	0.95	0.46	0.24	0.24	0.02	0.01	0.13	0.00	0.00	0.02	0.72	0.29	0.87	0.41	0.20	0.22	0.02	0.01	0.08	0.01	0.01	0.01
57	*te*	Elveden, ELV 95	5	0.78	0.40	1.03	0.47	0.26	0.24	0.01	0.01	0.03	0.00	0.01	0.01	0.71	0.29	0.80	0.41	0.21	0.24	0.02	0.01	0.05	0.01	0.01	0.01
58	*te*	Swanscombe	6	0.76	0.35	0.95	0.49	0.31	0.27	0.01	0.03	0.04	0.02	0.01	0.02	0.66	0.28	0.67	0.42	0.24	0.27	0.02	0.01	0.07	0.01	0.03	0.05
56	*te*	Barnham, BEF93	6	0.75	0.36	1.01	0.50	0.27	0.33	0.01	0.03	0.03	0.02	0.02	0.03	0.67	0.29	0.72	0.42	0.20	0.32	0.01	0.00	0.02	0.02	0.02	0.02
59	*te*	Woodston	4	0.77	0.40	1.01	0.53	0.30	0.29	0.01	0.01	0.02	0.01	0.01	0.01	0.66	0.30	0.69	0.42	0.23	0.30	0.01	0.01	0.04	0.01	0.01	0.01
55	*te*	Hoxne (2000, Stratum E)	4	0.77	0.38	0.96	0.49	0.28	0.31	0.01	0.02	0.13	0.02	0.01	0.03	0.69	0.29	0.75	0.42	0.23	0.29	0.00	0.01	0.01	0.01	0.01	0.01
60	*te*	**West Stow (Beeches Pit)**	4	0.78	0.37	0.98	0.49	0.27	0.30	0.01	0.01	0.03	0.01	0.01	0.01	0.71	0.31	0.79	0.42	0.25	0.29	0.01	0.01	0.03	0.01	0.01	0.01
61	*tr*	Dierden's Pit	2	0.81	0.38	1.06	0.51	0.27	0.19	0.01	0.02	0.03	0.01	0.01	0.01	0.71	0.28	0.72	0.43	0.22	0.19	0.01	0.01	0.05	0.01	0.01	0.00
55	*te*	**Hoxne (2001 (50); Stratum B2)**	4	0.76	0.33	0.96	0.48	0.28	0.30	0.01	0.01	0.12	0.03	0.02	0.01	0.69	0.32	0.76	0.43	0.24	0.29	0.01	0.01	0.05	0.01	0.01	0.01
62	*te*	Southfleet Road	7	0.78	0.39	1.02	0.52	0.30	0.27	0.01	0.04	0.01	0.01	0.02	0.01	0.68	0.30	0.72	0.43	0.22	0.27	0.02	0.01	0.07	0.01	0.01	0.02
61	*te*	Dierden's Pit	4	0.78	0.40	0.84	0.53	0.27	0.23	0.01	0.02	0.18	0.03	0.02	0.04	0.70	0.29	0.73	0.45	0.21	0.26	0.01	0.01	0.02	0.01	0.01	0.02
63	*te*	Clacton-on-Sea	4	0.78	0.38	0.98	0.53	0.33	0.28	0.01	0.02	0.08	0.02	0.01	0.02	0.69	0.26	0.70	0.46	0.25	0.30	0.02	0.01	0.1	0.01	0.02	0.01
64	*tr*	Waverley Wood	6	0.81	0.50	1.02	0.57	0.35	0.20	0.01	0.05	0.04	0.00	0.01	0.01	0.70	0.36	0.81	0.48	0.27	0.21	0.04	0.01	0.08	0.03	0.03	0.02
65	*te*	Sidestrand, Upper Unio-Bed	7	0.82	0.52	1.04	0.63	0.36	0.19	0.01	0.05	0.04	0.01	0.02	0.01	0.72	0.38	0.67	0.54	0.29	0.23	0.01	0.02	0.14	0.02	0.01	0.03
65	*te*	Sidestrand, Lower Unio-Bed	5	0.82	0.50	1.05	0.62	0.35	0.21	0.01	0.06	0.02	0.01	0.01	0.01	0.73	0.37	0.73	0.55	0.29	0.23	0.01	0.01	0.06	0.01	0.01	0.02
66	*tr*	Sugworth	6	0.84	0.53	1.13	0.65	0.39	0.14	0.01	0.02	0.05	0.01	0.02	0.01	0.75	0.37	0.75	0.56	0.28	0.15	0.01	0.01	0.11	0.01	0.01	0.01
67	*tr*	Little Oakley	2	0.87	0.51	1.11	0.65	0.40	0.13	0.00	0.00	0.02	0.01	0.01	0.00	0.76	0.36	0.73	0.56	0.28	0.16	0.00	0.00	0.01	0.00	0.00	0.00
68	*tr*	Pakefield, Unio-Bed	4	0.85	0.51	1.14	0.65	0.36	0.12	0.01	0.03	0.03	0.01	0.01	0.01	0.79	0.38	0.71	0.57	0.30	0.14	0.01	0.03	0.08	0.03	0.01	0.01
68	*tr*	Pakefield, laminated	3	0.84	0.52	1.06	0.64	0.45	0.12	0.01	0.05	0.03	0.02	0.03	0.01	0.77	0.46	0.78	0.58	0.35	0.14	0.01	0.02	0.05	0.01	0.02	0.01
69	*tr*	West Runton	11	0.86	0.52	1.12	0.67	0.40	0.13	0.01	0.01	0.04	0.01	0.01	0.01	0.79	0.39	0.87	0.61	0.33	0.14	0.02	0.02	0.06	0.02	0.01	0.01
70	*b/t*	**Bave**l	4	0.91	0.67	0.92	0.86	0.64	0.05	0.00	0.09	0.10	0.02	0.02	0.01	0.86	0.63	0.64	0.81	0.54	0.07	0.01	0.01	0.13	0.02	0.04	0.01
71	*tr*	Weybourne Crag	2	0.92	0.74	0.66	0.94	0.78	0.02	0.00	0.03	0.03	0.01	0.01	0.00	0.89	0.70	0.49	0.89	0.69	0.03	0.03	0.02	0.11	0.01	0.03	0.00
72	*tr*	**Tegelen**	4	0.94	0.82	0.71	0.93	0.83	0.01	0.00	0.11	0.15	0.01	0.01	0.00	0.92	0.81	0.42	0.90	0.75	0.02	0.01	0.02	0.06	0.01	0.03	0.00
71	*te*	Weybourne Crag	1	0.94	0.72	0.78	0.95	0.84	0.01	0.01	0.06	0.03	0.01	0.00	0.00	0.88	0.69	0.22	0.92	0.79	0.04	0.00	0.00	0.01	0.00	0.01	0.00
73	sp.	Thorpe Aldringham (Norwich Crag)	1	0.93	0.62	0.52	0.97	0.90	0.01	0.00	0.01	0.09	0.00	0.02	0.00	0.88	0.80	0.29	0.94	0.84	0.03	0.00	0.01	0.00	0.00	0.04	0.00
74	*te*	Frechen, Pliocene	4	0.95	0.93	0.41	0.95	0.96	0.01	0.01	0.12	0.18	0.01	0.02	0.00	0.94	0.94	0.28	0.93	0.91	0.02	0.01	0.01	0.11	0.01	0.02	0.01
75	*co*	Prospect Quarry (Eocene)	3	0.80	1.00	0.00	0.95	1.07	0.00	0.16	0.02	0.00	0.01	0.03	0.00	0.43	1.00	0.00	0.91	1.05	0.03	0.07	0.01	0.00	0.02	0.02	0.01

## Appendix A Supplementary material

Supplementary Fig. 1 Location map of the sites mentioned in the text from which *Bithynia* opercula have been analysed. The present courses of selected rivers discussed in the text are shown; lowland glaciation, especially in the Middle Pleistocene, will have affected the drainage pattern of these and other rivers. Sites are superimposed on a map (adapted from Crown copyright information supplied by the Met Office) of estimates of mean annual air temperature 1961–1990 (Perry and Hollis, 2005). Note that with the exception of the metropolitan heat islands almost all sites span a narrow range of temperature (9–10 °C). Sites with thermocouple data are highlighted in red (data given Penkman et al., 2011).
