# Supplementary figures and images for: An aminostratigraphy for the British Quaternary based on Bithynia opercula
Source: Quat Sci Rev. 2013 Feb 1;61:111–34. doi: 10.1016/j.quascirev.2012.10.046 (PMC3566634; doi:10.1016/j.quascirev.2012.10.046)

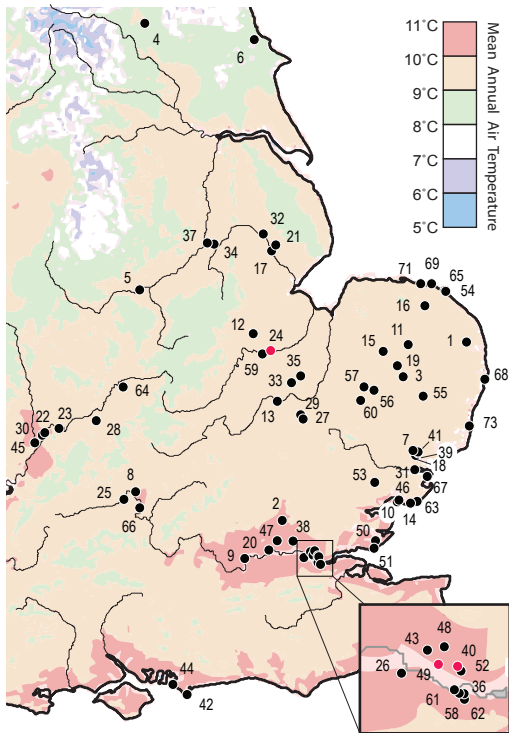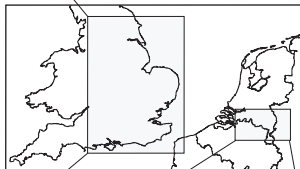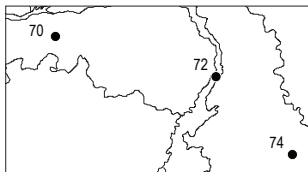

Supplement: Supplementary Fig. 1 — Location map of the sites mentioned in the text from which Bithynia opercula have been analysed. The present courses of selected rivers discussed in the text are shown; lowland glaciation, especially in the Middle Pleistocene, will have affected the drainage pattern of these and other rivers. Sites are superimposed on a map (adapted from Crown copyright information supplied by the Met Office) of estimates of mean annual air temperature 1961–1990 (Perry and Hollis, 2005). Note that with the exception of the metropolitan heat islands almost all sites span a narrow range of temperature (9–10 °C). Sites with thermocouple data are highlighted in red (data given Penkman et al., 2011). [file mmc1.pdf]
